# Predicting the Electron Requirement for Carbon Fixation in Seas and Oceans

**DOI:** 10.1371/journal.pone.0058137

**Published:** 2013-03-13

**Authors:** Evelyn Lawrenz, Greg Silsbe, Elisa Capuzzo, Pasi Ylöstalo, Rodney M. Forster, Stefan G. H. Simis, Ondřej Prášil, Jacco C. Kromkamp, Anna E. Hickman, C. Mark Moore, Marie-Hélèn Forget, Richard J. Geider, David J. Suggett

**Affiliations:** 1 Laboratory of Photosynthesis, Institute of Microbiology, ASCR (Academy of Sciences of the Czech Republic), Opatovický mlýn, Třeboň, Czech Republic; 2 Netherlands Institute of Ecology, Centre for Estuarine and Marine Ecology (NIOO-CEME), Yerseke, The Netherlands; 3 Centre for Environment, Fisheries & Aquaculture Science (CEFAS), Lowestoft, Suffolk, United Kingdom; 4 Finish Environment Institute (SYKE), Helsinki, Finland; 5 Ocean and Earth Science, University of Southampton, National Oceanography Centre, Southampton, Hampshire, United Kingdom; 6 Takuvic Joint International Laboratory, UMI 3376, Université Laval (Canada) CNRS (France), Department de Biologie and Québec-Océan, Université Laval, Québec City, Québec, Canada; 7 School of Biological Sciences, University of Essex, Colchester, Essex, United Kingdom; Royal Netherlands Institute of Sea Research (NIOZ), The Netherlands

## Abstract

Marine phytoplankton account for about 50% of all global net primary productivity (NPP). Active fluorometry, mainly Fast Repetition Rate fluorometry (FRRf), has been advocated as means of providing high resolution estimates of NPP. However, not measuring CO_2_-fixation directly, FRRf instead provides photosynthetic quantum efficiency estimates from which electron transfer rates (ETR) and ultimately CO_2_-fixation rates can be derived. Consequently, conversions of ETRs to CO_2_-fixation requires knowledge of the electron requirement for carbon fixation (*Φ_e,C_*, ETR/CO_2_ uptake rate) and its dependence on environmental gradients. Such knowledge is critical for large scale implementation of active fluorescence to better characterise CO_2_-uptake. Here we examine the variability of experimentally determined *Φ_e,C_* values in relation to key environmental variables with the aim of developing new working algorithms for the calculation of *Φ_e,C_* from environmental variables. Coincident FRRf and ^14^C-uptake and environmental data from 14 studies covering 12 marine regions were analysed via a meta-analytical, non-parametric, multivariate approach. Combining all studies, *Φ_e,C_* varied between 1.15 and 54.2 mol e^−^ (mol C)^−1^ with a mean of 10.9±6.91 mol e^−^ mol C)^−1^. Although variability of *Φ_e,C_* was related to environmental gradients at global scales, region-specific analyses provided far improved predictive capability. However, use of regional *Φ*_e,C_ algorithms requires objective means of defining regions of interest, which remains challenging. Considering individual studies and specific small-scale regions, temperature, nutrient and light availability were correlated with *Φ*_e,C_ albeit to varying degrees and depending on the study/region and the composition of the extant phytoplankton community. At the level of large biogeographic regions and distinct water masses, *Φ*_e,C_ was related to nutrient availability, chlorophyll, as well as temperature and/or salinity in most regions, while light availability was also important in Baltic Sea and shelf waters. The novel *Φ*_e,C_ algorithms provide a major step forward for widespread fluorometry-based NPP estimates and highlight the need for further studying the natural variability of *Φ_e,C_* to verify and develop algorithms with improved accuracy.

## Introduction

Accurately evaluating the impact of local environmental and global climate change upon trophic dynamics and biogeochemical nutrient cycling is fundamentally tied to how well primary productivity, defined here as carbon (CO_2_) fixation, is characterised. Following the incorporation of inorganic radio-labelled ^14^CO_2_ into algal cells has become a standard method for quantifying primary productivity. However, to this day, there still exists considerable uncertainty as to whether ^14^CO_2_ uptake measures gross primary productivity (GPP) or net primary productivity (NPP). Following the traditional view, GPP refers to carbon fixation without accounting for any carbon losses due to respiration and/or excretion, while NPP represents the carbon uptake rate after subtracting out any CO_2_ lost to oxidation of organic carbon over a diel cycle [1].

Of all global NPP, marine ecosystems account for ca. 50% [2], an amount equivalent to ca. 51·10^15^ g of fixed carbon per year [2], [3]; almost all of this productivity is from phytoplankton. However, marine ecosystem-scale productivity estimates contain a high degree of uncertainty, since they are ultimately derived by extrapolation from discrete measurements of NPP or GPP [4], [5] with limited spatial and temporal resolution. Remote sensing (ocean colour) productivity algorithms are the most widely used tool for making these extrapolations in order to better characterise the nature and extent of variability in NPP in marine ecosystems. However, these productivity algorithms are explicitly dependent on relatively few discrete, surface “truth” ^14^C primary productivity measurements. Many researchers have, therefore, turned to high resolution bio-optical-based approaches in order to meet this challenge.

Pulse Amplitude Modulated (PAM; [6]) and Fast Repetition Rate (FRR; [7], [8]) fluorometry provide the potential to dramatically increase the number of estimates of NPP in marine ecosystems [8]. As with other bio-optical sensors, active fluorometry can be utilised *in situ* and thus, measurements of productivity can be linked directly to measurements of physical/chemical variables at the time of sampling [9]–[11]. In addition, data can be collected at high temporal (seconds) and spatial resolution and/or over large scales [12]. If direct, *in situ* measurements of NPP using active fluorescence could be achieved, the advantages of this approach would represent a step change in operational capacity, in terms of accuracy and resolution, compared to ‘conventional’ ^14^C-based approaches, which are limited by the need to incubate relatively large water samples, often for long durations [13], with attendant potential problems due to ‘bottle effects’ [14], [15]. Thus, the widespread implementation of active fluorometers on broad-scale temporal or spatial sampling platforms, such as ships of opportunity and moorings would represent a major advance in evaluating the variability of primary productivity in seas and oceans.

Active fluorescence can be used to estimate the rate at which electrons flow from water through photosystem II to NADPH (the so-called linear photosynthetic electron transfer rate, ETR), and other electron acceptors. ETR is most closely related to the rate of gross O_2_ evolution, and potentially less directly to the subsequent production of energy (ATP) and reductant (NADPH) used to fix CO_2_. Thus, measurements of ETR by active fluorescence, at best, provide accurate estimates of the rate of gross O_2_ evolution from PSII [16], [17] and, hence, GPP. Unfortunately, many applications (e.g. climate models, fisheries assessments) require that primary productivity be expressed not as ETR of O_2_ evolution but rather in the photosynthetic currency (sensu Suggett et al. [18]) of fixed CO_2_, that is, NPP. As such, applicability of broad-scale active fluorescence-based measurements of ETR are potentially limited if ETRs cannot be easily converted to GPP and eventually NPP. Such conversion requires knowledge of the ETR to CO_2_ fixation ratio, i.e. the electron requirement for carbon fixation (*Φ_e,C_*) with units mol e^−^ (mol C)^−1^. With ETR and short-term (hours) CO^2^ fixation incubations representing GPP rather than NPP [19], [20], *Φ_e,C_* also captures gross rather than a net efficiency.

Since the introduction of active fluorescence to marine research, a number of studies have attempted to compare measurements of ETR with (quasi-)simultaneous measurements of CO_2_ uptake, the latter representing GPP, NPP or something in between depending on incubation times (1 hour to a full diel cycle) [10], [21]–[23] (Table 1, references therein). Initially, these exercises aimed to evaluate whether ETRs provided a robust quantification of GPP [32]–[35]. More recent studies have sought to understand the extent and nature of variation between the ETR and GPP and/or NPP [10], [11], [18], [36]. Numerous biochemical processes other than CO_2_ fixation can act to consume electrons, ATP and/or reductant; for example, the oxygenase activity of ribulose-1,5-bisphosphate carboxylase/oxygenase (Rubisco) via photorespiration [32], chlororespiration via a plastid terminal oxidase (PTOX) [33], [34], Mehler Ascorbate Peroxidase (MAP) activity [32] and nutrient assimilation [35], [37]. All these processes are expected to exhibit both taxon-specific and environmental dependencies with corresponding variability of *Φ_e,C_* [11], [18]; indeed, a recent compilation of published FRR-based *Φ_e,C_* data sets suggested the existence of some general taxonomic patterns in the variability of *Φ_e,C_* [38]. However, as yet, no single (global) systematic evaluation attempted to consider how environmental gradients regulate *Φ_e,C_*.

**Table 1:** Studies included in the meta-analysis.

Study/Cruise ID	Geographical area	Date	*σ_PSIIspec_*	*n_PSII_*	ETR/^14^C	N	References
AMT6	Atlantic	14 May–12 Jun 98	+	0.002	In situ^1^/PE^5^	40	[24], [25]
AMT11	Atlantic	15 Sep–09 Oct 00	+	0.003/0.002	In situ^1^/PE^5^	66	[24], [25]
AMT15	Tropical Atlantic	17 Sep–29 Oct 04	+	0.003	In situ^1^/PE^5^	16	[26]
D246	Celtic Sea	17–28 May 00	+	0.002	In situ^1^/PE^5^, SIS	7	[27], [28]
JR98	Celtic & Irish Sea	31 Jul–11 Aug 03	+	measured	On deck^1^/PE^5^	28	[11]
Time series	Gulf of Finland	11 Apr–15 Nov 00	+	0.002	In situ^1^/in situ^6^	19	[29]
Time series	Massachusetts Bay	27 Feb–29 Nov 00	1.75	‡	In situ^1^/PE^5^	37	[30]
Time series	Ariake Bay, Japan	26 Jun–04 Oct 06	1.75	0.002	In situ^4^/^13^C-SIS	2	[31]
SYNTAX	Baltic Sea	21 – 30 Jul 2010	n.a.	0.003	On deck^2^/FAST^act^	24	a
CEND0811	North Sea	08–12 May 11	n.a.	†	On deck^2^/FAST^act^	15	b
D366 UK-OA	UK & European shelf	06 Jun–12 Jul 11	+	†	On deck^2^/PE^5^	8	b
Time series	Bedford Basin, Canada	23 Feb–18 Apr 07	+	measured	On deck^2^/PE^5^	20	c
BIOSOPE2	Pacific Ocean	12 Sep–21 Nov 04	1.5	0.003	On deck^3^/PE^5^	25	d
SUPREMO11	Gulf of Finland	11–15 Apr 11	+	0.002	On deck^2,7^/PE^5^	42	e

Data were collected during cruises undertaken as part of the Atlantic Meridional Transect (AMT) Program, individual cruises to the Celtic Sea, Baltic Sea, North Sea, other UK and European shelf waters and the Pacific, as well as part of time series studies in the Gulf of Finland, Massachusetts Bay (USA), Ariake Bay (Japan) and Bedford Basin (Canada). Methodological differences existed in the way data were corrected for spectral discrepancies between the fluorometer and ^14^C-incubator light source (*σ_PSIIspec_*), in the estimates of the number of photosystem II (*n_PSII_*), and in the approach used to compare electron transport rates (ETR) and ^14^C fixation.

+ spectral corrections applied according to Moore et al. [11]. In some samples, a spectral correction factor of 1.75 was used while a spectral correction was n.a. denotes not applicable because primary productivity was measured on samples incubated in the cuvette holder of a FASTact Fluorometer.

*n_PSII_* was assumed to be constant (0.0033 or 0.0020 mol RCII (mol chl*a*)^−1^) or calculated either according to (‡) *n_PSII_* = 500 (*F_v_/F_m_*)/0.65 or (†) Oxborough et al.[7]. ETRs were either measured *in situ* or on discrete samples using a ^1^FAST^trecka^, or ^2^FAST^act^ FRR fluorometer, a ^3^FIRe benchtop fluorometer or a ^4^FRRF^Diving Flash^. Superscript ^5^ denotes photosynthesis irradiance curves measured on samples incubated for 1–4 hours using a photosynthetron [32] or equivalent incubator, ^6^ denotes 2 hour *in situ* bottle incubations, while ^7^ indicates rapid light curves carried out in on-deck laboratories under temperature controlled conditions. SIS stands for 24 hour on deck ‘simulated *in situ*’ incubations, and N is the number of observations for each study. a) Silsbe and Ylöstalo unpublished, b) Lawrenz, Capuzzo, Forster, Suggett unpublished, c) Suggett and Forget unpublished, d) Prášil, Gorbunov, Babin, Huot unpublished, e) Ylöstalo et al. unpublished.

Clearly, active fluorometry could become a much more powerful tool for estimating GPP (or even NPP) in carbon currency if generic relationships describing the dependency of *Φ_e,C_* upon routinely measured variables can be established. Therefore, we constructed a database of FRR-based measures of *Φ_e,C_* and associated environmental variables known to regulate primary productivity, (e.g. temperature, nutrients and light) from both previously published and unpublished data sets and used a meta-analytical approach to determine the predictability of *Φ_e,C_* from environmental variables. Specifically, we addressed the following three questions: (1) To what extent does *Φ_e,C_* vary within and between oceanic areas and water masses? (2) Can a ‘global’ *Φ_e,C_* ever be applied or should region-specific values always be used instead? (3) How is the variability of *Φ_e,C_* related to that of environmental factors or combinations thereof (light attenuation, nutrients, temperature, salinity, etc.)? Our analysis demonstrated that variability in *Φ_e,C_* was strongly correlated with the availability of light and nutrients, as well as temperature and/or salinity, albeit to varying degrees and depending on how data are organized into region-specific subsets. Predictive algorithms based on these relationships are presented. Treatment of data at the regional scale provided much improved predictive capability of these algorithms relative to large scale or global algorithms. Although many of the algorithms still need further verification and revision, this approach demonstrates strong relationships between *Φ_e,C_* and environmental gradients in many areas of the ocean.

## Materials and Methods

### Data compilation

**Figure 1:**
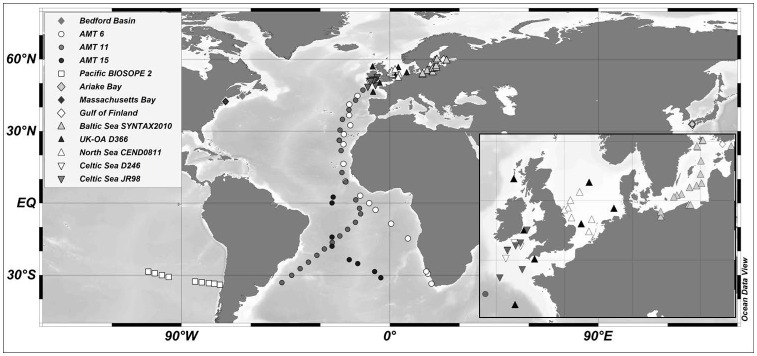
Data sets used in the meta-analysis. Gulf of Finland includes both the SUPREMO2011 and the Raateoja et al. [29] study (See Table 1 for details).

A comprehensive assessment of the variability in *Φ_e,C_* from published literature and previously unpublished data was performed. Web of Science and JSTOR search engines were used to retrieve published data on coincident FRR fluorescence-based ETRs and carbon fixation rates from marine habitats, yielding 17 studies. However, only 7 of these studies reported key environmental variables in addition to *Φ_e,C_* or *both* ETRs and CO_2_-fixation rates (resembling either NPP or GPP depending on the incubation lengths and growth conditions), from which *Φ_e,C_* could be calculated. Previously unpublished data corresponding to parallel ETR and CO_2_ fixation measurements from an additional 7 field campaigns in 2004–2011, some of which were undertaken as part of PROTOOL (http://www.protool-project.eu/), were also included (Table 1). Together, these data sets included ten research cruises and four time series studies covering a range of different geographical areas (Fig. 1), including the temperate, tropical and subtropical Atlantic Ocean (AMT6, AMT11, AMT 15), Massachusetts Bay (USA), Bedford Basin (Canada), Ariake Bay (Japan), the Celtic and Irish Sea (D246, JR98), the North Sea (CEND0811/PROTOOL, Gulf of Finland (including SUPREMO11/PROTOOL), the Baltic Sea (SYNTAX 2010/PROTOOL), UK and European shelf waters (D366/PROTOOL) and the Pacific Ocean (BIOSOPE).

A comprehensive data matrix with 333 different samples and their corresponding physico-chemical and methodological variables was created. Physico-chemical variables included salinity, temperature, nutrient concentrations (NO_3_^−^ and PO_4_^3−^), the diffuse vertical attenuation coefficient (*K_d_*) of photosynthetically active radiation (PAR), optical depth (ζ) and chlorophyll *a* (chl*a*), with the latter being used as a proxy for phytoplankton biomass. We fully acknowledge that other environmental variables, such as the silicate or iron are often also key in determining phytoplankton community composition and physiological responses and, hence, influence *Φ_e,C_*. However, these data were either not collected as part of the studies included here or not available. Methodological variables included differences in FRR protocols and ^14^C incubation techniques (see below). Locations of samples were characterized by latitude and longitude, while seasonal differences were characterized by converting sampling dates to Julian day. All the environmental data were either kindly provided by the authors of the published work, the British Oceanographic Data Centre (BODC, www.bodc.ac.uk) or were taken from the original publications and digitized (Plot Digitizer 2.5.0, Free Software Foundation) from relevant figures.

Values of *K_d_* (in units of m^−1^) were derived from vertical irradiance profiles for the AMT cruises, Bedford Basin, the Celtic Sea (JR98), Baltic Sea (SYNTAX2010), Ariake Bay, the Gulf of Finland [29], the UK-Ocean Acidification cruise (D366) and the Pacific Ocean (BIOSOPE2) dataset.

For the remaining studies, MODIS 4 km satellite products of euphotic zone depth (z_eu_), here defined as 1% of surface PAR, produced by the Giovanni online data system (NASA Goddard Earth Science Data and Information Services Center) averaged over 8 days were used to calculate *K_d_* by solving 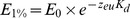
(1)for *K_d_* with E_0_ set to 100%. Multiplying *K_d_* by the actual sampling depth then gives *ζ* (dimensionless), with *ζ*<4.6 corresponding to irradiance levels >1% [39]. For data collected prior to 2002 (Massachusetts Bay), SeaWIFS 9 km z_eu_ products were used for calculating *K_d_* and subsequently *ζ*.

### FRRF measurements

FRR fluorescence transients were either measured *in situ* or on discrete samples on-board using FAST^tracka^ I or FAST^tracka^ II fluorometers with FAST^act^ systems (Chelsea Technologies Group, Ltd., West Molesey, UK), FIRe benchtop instruments (Satlantic, LP, Halifax, Canada) or a FRR^Diving Flash^ fluorometer (Kimoto Electric Co., LTd., Osaka, Japan) (see Table 1). FRR fluorometers were routinely programmed to generate a standard protocol with 50–100 single turnover (ST) saturation flashlets of 1.1–3.3 µs duration at 1–3.6 µs intervals [18], [40]. Each induction curve was separated by ∼10 ms. To increase signal to noise, between 5 and 160 sequential induction curves were averaged per acquisition.

The biophysical model of Kolber et al. [41] was fitted to all fluorescence transients to derive the initial fluorescence (*F_0_*) and maximal fluorescence (*F_m_*) yields measured in the dark, the minimal (*F_0_'*), steady state (*F'*) and maximal (*F_m_'*) fluorescence yield measured under ambient irradiance, as well as the functional absorption cross section of photosystem II (PSII) in the dark (*σ_PSII_*) and light (*σ_PSII_'*) (in units of Å^2^ quanta^−1^). For most studies, the photosynthetic electron transfer rate through PSII (units of mol e^−^ (mg chl*a*)^−1^ h^−1^) [42], [43] was calculated as: 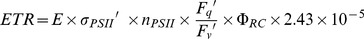
(2)where E is light intensity, *n_PSII_* the ratio of functional PSII reaction centres to chlorophyll (in units of mol RCII (mol chl*a*)^-1^), *Φ_RC_* an assumed constant of 1 electron yielded from each reaction centre II (RCII) charge separation and 2.43 · 10^-5^ is the factor that accounts for the conversion of Å^2^ quantum^-1^ to m^2^ (mol RCII)^-1^, mol chl*a* to mg chl*a*, seconds to hours and μmol quanta to mol quanta [13], [29]. Measurements of *σ_PSII_'* account for transient non-photochemical quenching in the antenna bed as a result of exposure to transient light [44], and thus, values of *σ_PSII_'* were typically taken from the FRRf dark chamber to increase signal to noise.

The PSII efficiency factor, termed *F_q_'/F_v_'* (was calculated as (*F_m_'–F'*)/(*F_m_'–F_0_'*) either from fluorescence emissions measured sequentially on dark acclimated samples and then under actinic light, or estimated as the difference in the apparent PSII photochemical efficiency between the FRR light and dark chamber quasi-simultaneously, as in Suggett et al. [25]. In this case, no blank correction to the fluorescence yields is required because any contribution of background fluorescence (to *F’*, *F_0_’* or *F_m_’*) effectively cancels between light and dark chambers/conditions [25], [44]. For two studies [25], [28], the electron transport rate was evaluated using the equivalent equation:



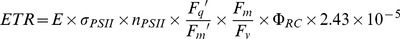
(3)where values of *F_v_/F_m_* and *σ_PSII_* are measured in the dark or assumed dark values based on measurements from deeper in the water column, that is, from depths where E<E_K_ and where non-photochemical quenching can be assumed to be negligible [25]. In the latter case, the influence of both photochemical and non-photochemical quenching are all accounted for by changes in *F_q_’/F_m_’*. This slightly alternative approach was employed to enable ETRs to be estimated in the absence of a 'dark chamber', and consequently, corresponding light and dark acclimated samples from all measurement depths, which would be required in order to apply Eq. 2. Both, Eqns. 1and 2, appear to return consistent estimates for quenching [8], and hence, ETR. It should further be noted that derivation of *F_q_'/F_v_'* requires a measurement to be made following brief dark exposure. Consequently, under circumstances where rapid reversal of certain components of NPQ occurs during the dark measurements, *F_q_'/F_v_'* may be overestimated potentially causing overestimates of ETR evaluated using Eqn. (2) by up to 30% in phytoplankton cultures [8].

Apart from the blank correction of the absolute fluorescence yields (see above and [45]), the accuracy of the ETR also depends on how variable some assumed ‘constants’ are and how well certain corrections are applied; specifically, (1) *n_PSII_* [46], (2) the spectral correction of *σ_PSII_*, and (3) differences between ETR-based and CO_2_-based values of light harvesting efficiency (*α*) and maximum photosynthesis rates (P_max_). Firstly, *n_PSII_* is known to vary between taxa and environmental conditions by up to a factor of 5 [8] but is rarely measured in the context of FRR-based productivity studies. In fact, of the studies used here only Moore et al. [11] and Suggett & Forget (unpubl.) used direct measurements of *n_PSII_*. The majority of the other studies assumed values of 0.002 and 0.003 mol RCII (mol chl*a*)^−1^ for populations dominated by eukaryotes and prokaryotes, respectively [47], or calculated *n_PSII_* using a newly developed algorithm, which will also have associated caveats [7]. In evaluating the use of this assumed constant *n_PSII_* in some of our data sets, we compared *Φ_e,C_* derived with constant *n_PSII_* to *Φ_e,C_* based on measured *n_PSII_* (see discussion). We return to the issue of spectral corrections in the section “matching ETRs with C-uptake”.

Although the FRRf approach provides a general estimate of *α* and P_max_, the light saturation point (E_K_) for the ETR may be lower than that from ^14^C uptake because of, for example, electron consuming processes [48]–[50]. The latter can cause the turnover time for QA to decouple from that of the whole chain PSII turnover, so that the E_K_ for the ETR may not be wholly indicative of where light is limiting or saturating for CO_2_ uptake.

### ^14^CO_2_ fixation rates

CO_2_ fixation was measured by either *in situ* incubations [29], simulated *in situ* incubations [27], [28], [30], [31] or photosynthesis versus irradiance (PE) relationships [11], [25], [26] in a ‘photosynthetron’ according to Lewis and Smith [51] or an equivalent thereof (see Table 1 for details). CO_2_ fixation in Ariake Bay (Japan) was measured using the stable isotope ^13^C-labelled NaH^13^CO_3_ [31]. Incubation lengths varied between the different studies, with the vast majority incubating for a few (1–4) hours and thus capturing productivity somewhere between GPP and NPP [19], [20].

### Matching ETRs with C-uptake

Ideally, measurements of photosynthetic carbon fixation and ETR should be made simultaneously on the exact same sample to reduce errors arising from differences in sample treatment and handling [8], [18]. Moreover, the methods used to determine CO_2_ fixation differed considerably between studies (Table 1). Some of the most recent studies in the Baltic Sea (SYNTAX2010) and North Sea (CEND0811) followed recommendations of Suggett et al. [8] and measured ^14^C uptake by placing the radioactive sample directly in the cuvette holder of the FASTact fluorometer. This simultaneous incubation technique avoided discrepancies in the intensity and spectral quality of the actinic light sources. In this way, ETR and GPP were measured for 1 h on the same sample at *in situ* temperatures and at a light intensity corresponding to either half or twice the value of E_K_ (as determined from FRRf rapid light curves); these light intensities relative to E_K_ were chosen to yield a measure of *Φ_e,C_* corresponding to an irradiance for light-limited and light saturated photosynthesis.

For most studies, ^14^C-specific PE experiments were performed on discrete water samples whilst FRR data (and hence ETRs) were determined from *in situ* casts or on deck (Table 1). Incubation lengths varied between the different studies, with the vast majority incubating for a few (1–4) hours and thus capturing productivity rates somewhere between GPP and NPP [19], [20]. For this same reason, adhering to strict definitions of NPP and GPP throughout the manuscript is not always possible. We have therefore specified NPP or GPP where possible but otherwise use the terms CO_2_ fixation or primary productivity. *In situ* FRR data were compared to the PE data from the same depth as the discrete water sample. For on-deck based ETR measurements during SUPREMO11, actinic light was provided by an external programmable light source. The light intensity measured during the FRR data acquisition (used to calculate the ETR) was then applied to the ^14^C-PE equation to yield a measure of the instantaneous ^14^C uptake for matching with the ETR. For some studies, simulated *in situ* ^14^C data were collected (corrected using ^14^C-PE to yield ‘gross’ ^14^C uptake [27], [28] (Table 1). Here, we adopted a similar approach but further normalised daily ^14^C uptake rates to hourly rates based on knowledge of the light regime.

Direct comparison of the ETRs and ^14^C uptake requires that the spectral quality of the actinic light of the FRRf and ^14^C incubator is either equivalent or corrected for. The spectral values of *σ_PSII_* (measured with a blue LED) also needed to be scaled to the light quality of the actinic source used for the ^14^C incubations [40]. In most cases, *in situ* FRR values of *σ_PSII_* and the ^14^C actinic light source were both spectrally corrected to match the spectral quality corresponding to the sample depth [40]. In cases where both FRR- and ^14^C- PE curves were measured on discrete samples, data were spectrally corrected following Moore et al. [11]. For the studies that did not employ a spectral correction (Table 1) we assumed a constant factor of *σ_PSII_*/1.75 [11], [31] and *σ_PSII_*/1.5 (Prášil et al. unpubl.) based on approximate values from the other studies for the water types in question.

Taking into account the light history of samples from different studies was the greatest challenge in compiling the database due to considerable inconsistencies in the availability and quality of light data. Thus, irradiances were expressed as optical depths, and instantaneous light was standardised by normalizing E relative to the saturating light intensity, E_K_, as determined from ^14^C-specific PE curves, i.e. E∶E_K_ (dimensionless). In this way, E can be considered relative to the light history to which cells are acclimated [52] providing information as to whether the values of *Φ_e,C_* correspond to light limited photosynthesis (E∶E_K_<1) or light saturated photosynthesis (E∶E_K_>1). Note, however, that E comes from *in situ* PAR measurements, while E_K_ was usually based on measurements made in the laboratory. In this case, the spectral quality of E and E_K_ may differ and values of E/E_K_ ratios from different studies must be treated with caution.

All CO_2_ fixation rates (in mg C L^−1^ h^−1^) were normalised to the corresponding chl*a* concentration to yield CO_2_ uptake rates as mol CO_2_ (mg chl*a*)^−1^ h^−1^; thus values of the *Φ_e,C_* (in mol electrons mol CO_2_^−1^) were determined as
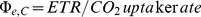
(4)


Given the large array of data sets and the various differences in approach for determining both ^14^C uptake and FRR fluorescence ETRs [8], [18], we recognise that a major assumption inherent to our analysis is that environmental influences outweigh methodological influences on *Φ_e,C_*, both, within and between studies. Fortunately, all studies employed similar FRRf protocols (above). Even so, information on core variables associated with the ETR determinations were initially included in our database and examined alongside the environmental variables to verify any role of method upon apparent variability in *Φ_e,C_* (see “statistical approach” section). Note that the ‘methodological’ data across studies is categorical rather than continuous, (e.g. spectral correction of *σ_PSII_* applied or not applied); therefore, the available information was converted into a Boolean code with numbers one and zero representing use and non-use of a particular method, respectively. In total, 3 methodological variables were ultimately included in the initial data set: *n_PSII_* assumed or measured, spectral correction of *σ_PSII_* assumed or measured, and E∶E_K_>1 or E∶E_K_<1.

At this point it should also be noted that apart from environmental factors, differences in phytoplankton community composition may also influence *Φ_e,C_*. Unfortunately, taxonomic data was not consistently available for the majority of studies. However, we assume that phytoplankton community composition will partially reflect environmental characteristics and some influence of community composition on *Φ_e,C_* will likely be implicitly accounted for in our analyses purely based on environmental variables.

### Statistical approach

Many of the following analyses were carried out on both the entire dataset or on individual subsets thereof, which, for example, represent different oceanic regions. Although the majority of our samples were collected in the Atlantic Ocean and adjacent shelf waters while other regions (e.g. Pacific, Indian Ocean, and Mediterranean Sea) are underrepresented/not represented at all, we still use the term 'global' for analyses carried out on the dataset as a whole.

Spearman Rank Order Correlation analysis on the individual data sets (i.e. samples grouped by study) were used to identify key variables that may be associated with *Φ_e,C_*. Correlations, evaluated in SPSS 15.0 (SPSS Inc., Chicago, IL, USA) were considered significant when *p*<0.05. These correlations were carried out i) on the individual data sets (i.e. samples grouped by study), ii) on the global dataset, iii) on studies pooled according to regions (e.g. North Sea, Baltic Sea, Atlantic Ocean etc.) and iv) according to shelf/oceanic waters, thus providing an overview of which variables may be important. All data were then combined into one large database to carry out all other statistical analyses using non-parametric multivariate techniques in PRIMER-E version 6 (PRIMER-E, Ltd. Ivybridge, Devon, UK) [53], unless noted otherwise.

Inter-correlated and right-skewed physico-chemical variables (e.g. salinity, temperature, chl*a*, NO_3_−, and PO_4_^3−^) were identified using Draftsman plots and then square-root transformed to stabilise the variance and ensure that Euclidean distance could be used as an appropriate similarity measure (see below). To account for differences in scales and units between different variables, the latter were normalized by subtracting the mean from each entry of a single variable and dividing it by the variable's standard deviation.

Non-metric Multidimensional Scaling (nMDS) was used as a means to map environmental characteristics of samples in a low-dimensional space based on a triangular resemblance matrix that was created by calculating Euclidean distances between every possible pair of samples. Hence, Euclidean distances between samples represented the dissimilarities in the suite of their physico-chemical properties. Principal Component Analysis (PCA) was then used to identify variables accounting for the differences between samples. Variables initially included in the PCA were salinity, chl*a*, NO_3_^−^, PO_4_^3−^, temperature, *K_d_*, and *ζ*, as well as a Boolean-matrix of methodological differences with regard to *n_PSII_*, *σ_PSII_* and E∶E_K_ (as described above, Table 1).

Inclusion of the methodological and location variables (latitude, longitude (absolute values) and Julian day) usually increased dissimilarities, i.e. Euclidean distances, between samples of different studies compared to analyses from which methodological and location variables had been excluded. Because the ‘methodological choices’, in particular, cannot be effectively incorporated into predictive algorithms for *Φ_e,C_*, the results we show in the main text here are based on PCAs without methodological and location variables. Nevertheless, the results of the PCA including these methodological and location variables have been included in Appendix S1 (Fig. S1). Only those variables with the highest eigenvectors were included in any further analysis to assess the variability in *Φ_e,C_*.

Both nMDS and PCA generate low dimensional ordinations. Here we only show the nMDS plots, which matched the PCA plots well, because nMDS better preserves the distances between samples when mapping them onto a 2D or 3D space [53]. In addition nMDS provides a measure, the stress value, of how well the low dimensional ordinations represent the distances between samples. In all cases the stress value of the nMDS was considerably lower in the 3D ordination relative to the 2D ordination. Thus, the 3D-images are presented here where the individual planes (x-y, x-z, y-z) are separate panels.

Because environmental factors showed considerable spatial and temporal variation, which, in turn, influence physiological responses of phytoplankton and subsequently *Φ_e,C_* to varying degrees, hierarchical agglomerative cluster analysis and a similarity profile (SIMPROF) test were used to find and define groups of samples with similar physico-chemical properties [53]. SIMPROF tests were used as a stopping rule of the cluster analysis, so that successive partitions along the branches of the dendrogram are only permitted if the null hypothesis of ‘no structure between samples’ was rejected. To reduce the number of clusters to a manageable size and to ensure that a sufficient number of samples large enough for algorithm development were included in each cluster, the *p*-value for the SIMPEROF test was reduced to 0.005. Thus, once a non-significant test result was obtained (*p*>0.005), samples below that similarity level were no longer partitioned into clusters and could be regarded as homogeneous [53].

All data were then re-grouped according to the results of the cluster analysis and SIMPROF test. The PRIMER-BEST match permutation test was then used to identify variables and variable combinations that best “explain” the variability in *Φ_e,C_* [53]. That is, the algorithm randomly permutes a resemblance matrix generated from *Φ_e,C_* (based on all possible pair-wise sample combinations) relative to a resemblance matrix generated from subsets of the environmental data matrix searching for high rank correlations between the two and generating a correlation coefficient (ρ). Repeated (99) permutations of randomly ordered environmental data follow to test the significance of the results at a level of *p*<0.01 [53].

One of the main goals of this meta-analysis was to generate algorithms to predict *Φ_e,C_* from environmental variables for specific regions. Therefore, mathematical relationships between the environmental data and *Φ_e,C_* were produced for each cluster that contained at least 5 data points using multiple linear regression (MLR). Only those variables and variable combinations that were significantly correlated with *Φ_e,C_* according to the PRIMER-BEST-test were entered into the MLR in SPPS.

## Results

### Spatial and temporal variability in *Φ_e,C_*

**Figure 2:**
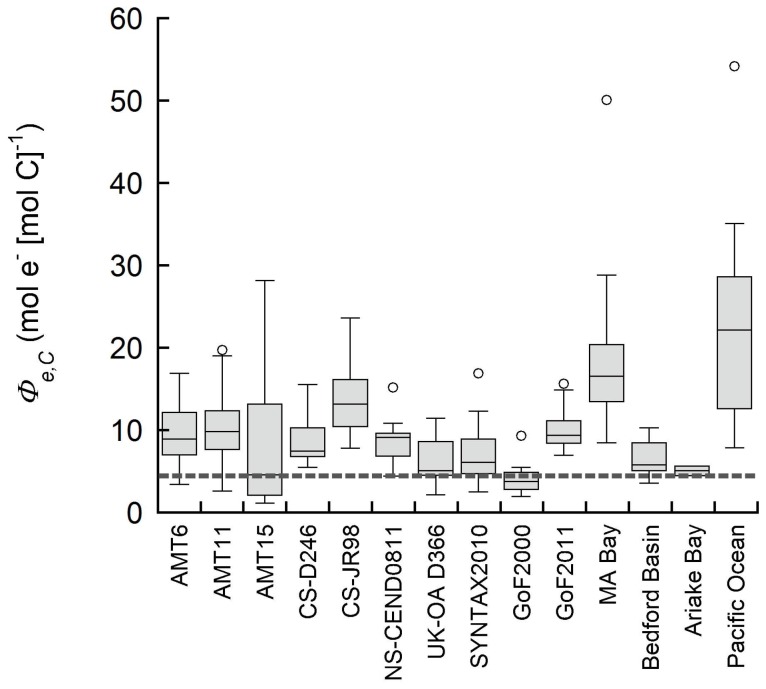
Variability in the electron requirement of carbon fixation (*Φ_e,C_*) derived from corresponding FRRf-ETR and ^14^C-primary productivity measurements. Boxes represent the median, 0.25 and 0.75 quartile, whiskers are the 1.5 interquartile range. Outliers are indicated by open circles. The dashed grey line is the theoretical reference ratio of 4 mol e^−^ [mol C]^−1^. AMT  =  Atlantic Meridional Transects, CS  =  Celtic Sea, NS  =  North Sea, GoF  =  Gulf of Finland 2000 (time series), UK-OA  =  UK Ocean Acidification cruise, MA  =  Massachusetts Bay (time series). SYNTAX2010 is a Baltic Sea cruise, GoF2011 is the SUPREMO2011 study and the Pacific Ocean study is the BIOSOPE- cruise to the Southeast Pacific (see Table 1, Figure 1 for details).

Mean values for *Φ_e,C_* in all but four studies were <10 mol e^−^ (mol C)^−1^, resulting in a global mean (± standard deviation) of 10.9±6.91 mol e^−^ (mol C)^−1^. Values of *Φ_e,C_* less than the theoretical ratio of 5 mol e^−^ (mol C)^−1^ (see discussion) were observed in the Gulf of Finland 2000 and in part of the AMT 15 data. Variability of *Φ_e,C_* within and between individual studies was considerable, with a total range of 1.15–54.2 mol e^−^ (mol C)^−1^ for all studies combined (Fig. 2). Within-study variation of *Φ_e,C_* was largest in the Pacific Ocean (7.9–54.2 mol e^−^ (mol C)^−1^), Massachusetts Bay (8.5–50.1 mol e^−^ (mol C)^−1^), and the AMT 15 data (1.1–28.2 mol e^−^ (mol C)^−1^); in contrast, least within-study variance was typically observed for time series' at a single location (Ariake Bay: 5.1–5.7 mol e^−^ (mol C)^−1^, Bedford Basin: 3.6–10.3 mol e^−^ (mol C)^−1^, and the Gulf of Finland 2000: 2.0–9.4 mol e^−^ (mol C)^−1^). As there were no distinct differences in the number and types of *σ_PSIIspec_* and *n_PSII_* corrections between the time series studies and the other cruises, it would appear that variability of *Φ_e,C_* is generally greater spatially than temporally within the included locations. The time series studies included here were of relatively short duration (1.5–7 months), however, temporal variability may further increase if long-term studies (multiple years) are included.

Spearman Rank Oder Correlations could be found between *Φ_e,C_* and every environmental variable included in the study, albeit to varying degrees and depending on how data were grouped. On a global scale, i.e. combining the entire data set, *Φ_e,C_* exhibited significant positive correlations with Julian day (i.e. time/season) and salinity, while significant negative correlations existed with latitude, longitude, chl*a* concentrations *K_d_* and ζ (Table 2). These correlations explained at least 12% of the variance in all cases. To consider if the global data pool may obscure regional-scale trends, correlations were also carried out (i) on the individual studies and (ii) by pooling studies representative of particular regions, such as, the Baltic Sea (including the Gulf of Finland), the Atlantic Ocean (all AMT cruises combined) and by combining data from shelf and oceanic waters, respectively.

**Table 2:** Spearman Rank Order Correlation Coefficients for correlations between *Φ_e,C_* and environmental variables.

	Lat	Lon	JD	Depth	Temp	Sal	NO_3_^−^	PO_4_^3−^	Chla	K_d_	ζ
Studies combined											
All Studies	−0.272	−0.450	0.297	-	-	0.226	-	-	−0.130	−0.244	−0.123
Shelf	−0.431	−0.546	0.214	0.149	-	0.376	0.229	0.186	−0.136^a^	−0.511	−0.155
Oceanic	−0.324	−0.320	−0.201	-	-	-	−0.243	−0.167^a^	-	-	-
AMT combined	-	−0.212	-	-	0.250	0.276	−0.361	−0.417	−0.175^a^		
Baltic combined†	-	0.347	0.530	−0.337	0.271	−0.438	0.276	0.269	0.320	−0.562	−0.569
AMT 6	0.322	-	0.322	-	0.520	-	−0.374	−0.406	−0.577	−0.415	-
AMT 11	−0.344	−0.431	0.344	-	-	0.329	-	−0.338	-	-	-
AMT 15	-	-	-	−0.709	-	-	−0.765	-	−0.681	-	−0.756
CS-D246	-	-	-	-	-	-	-	-	-	-	-
CS-JR98	-	-	-	−0.564	-	-	−0.391	-	-	-	−0.482
CS-DS46+JR98	-	-	0.377	−0.614	0.423	-	-	-	−0.374	-	−0.416
PO-BIOSOPE	-	-	-	-	-	-	-	-	-	-	−0.482
NS-CEND0811	-	-	-	0.616	-	-	-	-	-	-	-
UK-OA D366	-	-	-	-	-	-	-	-	-	-	-
SYNTAX2010	-		-	-	0.568	-	-	-	-	-	-
GoF2000	n.a.	n.a.	-	n.a.	-	-	-	-	-	-	-
SUPREMO11	-	-	-	−0.645	-	-	−0.494	-	-	-	−0.653
GoF +SUPREMO11	0.352	0.752	−0.737	−0.310^a^	−0.683	0.668	0.582	0.586	−0.452	−0.705	−0.684
Bedford Basin	n.a.	n.a.	-	n.a.	-	0.582	-	-	-	-	-
MA Bay	-	-	-	-	-	-	-	-	-	-	-

Values in bold or normal font indicate significant correlations where *p*<0.01 and *p*<0.05, respectively.

JD is Julian Day. † denotes Baltic proper and the Gulf of Finland combined. - non-significant correlations (*p*>0.06). ^b^ denotes correlations with 0.05>*p*>0.06.

At the level of the individual study, different environmental variables showed varying degrees of correlation depending on the region. Multiple notable trends were evident between *Φ_e,C_*, sampling depth, *K_d_*, ζ and nutrient availability, including: 1) changes of *Φ_e,C_* with depth, which were observed during AMT15, in the Celtic Sea, the North Sea (NS-CEND0811) and the Gulf of Finland/Baltic Sea; 2) a decline in *Φ_e,C_* with increasing *K_d_* during AMT6 and in the Baltic Sea, and with increasing *ζ* during AMT15, in the Celtic Sea, Pacific Ocean and in the Gulf of Finland/Baltic Sea; and 3) a change in *Φ_e,C_* with nutrient concentrations, during the AMT cruises, one of the Celtic Sea cruises (JR98) and in the Gulf of Finland/Baltic Sea. In the Baltic Sea (SYNTAX2010) and Bedford Basin significant correlations existed between *Φ_e,C_* and temperature and salinity, respectively. No correlation of *Φ_e,C_* with common environmental variables were found in Massachusetts Bay or during the UK-OA cruise. Note also, that depth-dependent trends in *Φ_e,C_* could only be assessed where multiple depths had been sampled at each station across large proportions of the cruise transect/time series, such as in the open ocean studies where data were available up to a depth of ∼200 m (e.g. AMT cruises, Pacific Ocean, Celtic Sea JR98) or the Gulf of Finland (SUPREMO11) studies where up to four depths were sampled across the euphotic zone at each station. Thus, a lack of relationship between *Φ_e,C_* and depth may simply reflect limited sampling depths or, in shallow waters with rapid vertical mixing, acclimation to an average water column irradiance.

**Figure 3:**
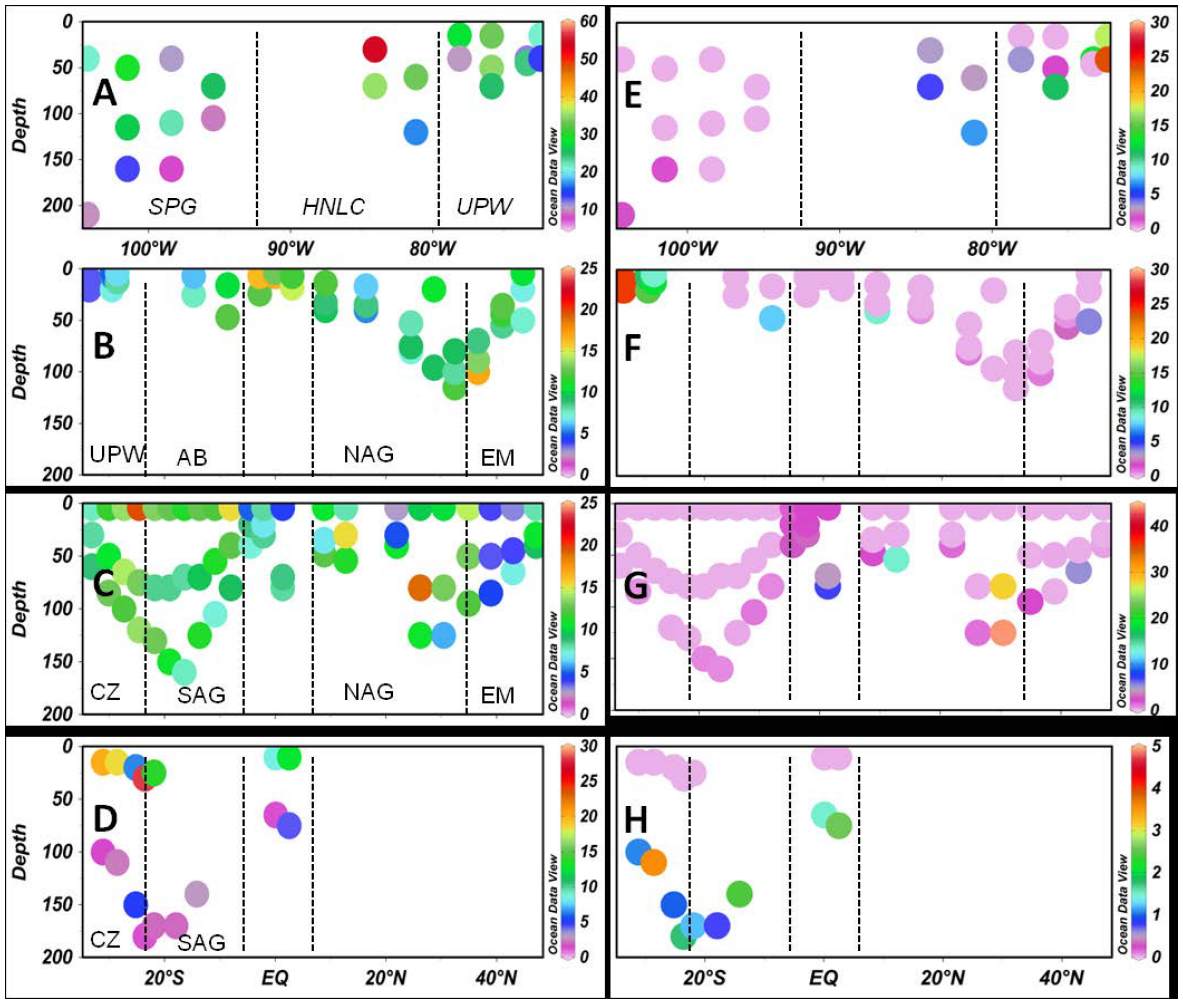
Changes in the electron requirement of carbon fixation (*Φ_e,C_*) and NO_3_^−^ across 4 cruise transects. (A) and (E) Pacific Ocean (BIOSOPE), (B) and (F) AMT 6, (C) and (G) AMT 11, and (D) and (H) AMT 15. Units of *Φ_e,C_* (left panels) are in mol e^−^ (mol C)^−1^), NO_3_^−^ concentrations (right-hand panels) are in µmol L^−1^. Dashed lines denote transitions between different biogeographical provinces, where SPG is the South Pacific Gyre, HNLC High Nutrient Low Chlorophyll areas, UPW denotes upwelling regions, AB is the Angola Basin, SAG South Atlantic, NAG the North Atlantic Gyre, EM the Eastern Margin off Western Europe, and CZ is the Suptropical Convergence zone. Note, difference in scales on both axes and the colour contours.

A notable feature for the Pacific and Atlantic transects as well as the Celtic Sea (JR 98, data not shown) was a pronounced increase of *Φ_e,C_* surface waters with low NO_3_^−^ and/or PO_4_^3−^ availability (Fig. 3, Table 2). In fact, the highest *Φ_e,C_* values across all studies corresponded to a surface water lens in the HNLC and Humboldt upwelling region of the Pacific Ocean. For the other Atlantic studies, AMT 6 and 11, *Φ_e,C_* also declined with increasing NO_3_^−^ and/or PO_4_^3−^, but as the depth of the surface mixed layer and/or nitracline varied between stations, the relationship between depth and *Φ_e,C_* was not linear.

Negative correlations between *Φ_e,C_* and ζ for AMT15, the Celtic Sea (JR98), the Pacific Ocean and the Gulf of Finland indicated that *Φ_e,C_* often increased towards depths of greater light availability in surface waters or with declining *K_d_* values from coastal to offshore regions. Negative correlations of *Φ_e,C_* with depth, *K_d_* and ζ were also found but only in Baltic Sea and shelf waters.

### Regional differences in environmental conditions

The initial correlative exercise for each separate study demonstrated that the relationships between *Φ_e,C_* and environment are dependent upon how data within and between data sets are grouped; therefore ‘choice’ of grouping will inevitably influence the outcome of empirical algorithms generated to predict *Φ_e,C_* from physico-chemical variables. A major objective of this study was to identify environmental predictors of *Φ_e,C_*; therefore PCA in combination with cluster analysis was subsequently used to 1) identify the principal differences in environmental condition between sites regardless of the study to which they belonged and 2) to form clusters of sites with similar environmental conditions, which could then be analysed further with respect to their association with *Φ_e,C_*.The first three principal components of the PCA combined accounted for 86% of the cumulative variation in environmental variables (PC1 47%, PC2 24% and PC3 15%). Temperature, chl*a* and *K_d_* had the highest coefficients for the linear combination of variables comprising PC1, i.e. they were associated with the separation of samples along PC1 (Table 3). Salinity, NO_3_^−^ and PO_4_^3−^ differentiated samples along PC2, while a single variable, ζ, had the highest Eigenvectors of PC3.

**Table 3:** Eigenvectors of Principal Component Analysis (PCA).

Variable	PC1	PC2	PC3
Temperature	−0.476	0.111	−0.184
Salinity	−0.325	−0.509	0.054
NO_3_^−^	0.337	0.541	−0.087
PO_4_^3−^	0.399	0.491	−0.020
Chla	0.464	0.232	0.020
K_d_	0.424	0.373	−0.091
ζ	0.001	0.023	−0.973

Values in bold represent variables with the highest coefficients for each PC. *K_d_* is the vertical attenuation coefficient of photosynthetically available radiation and *ζ* is optical depth.

**Figure 4:**
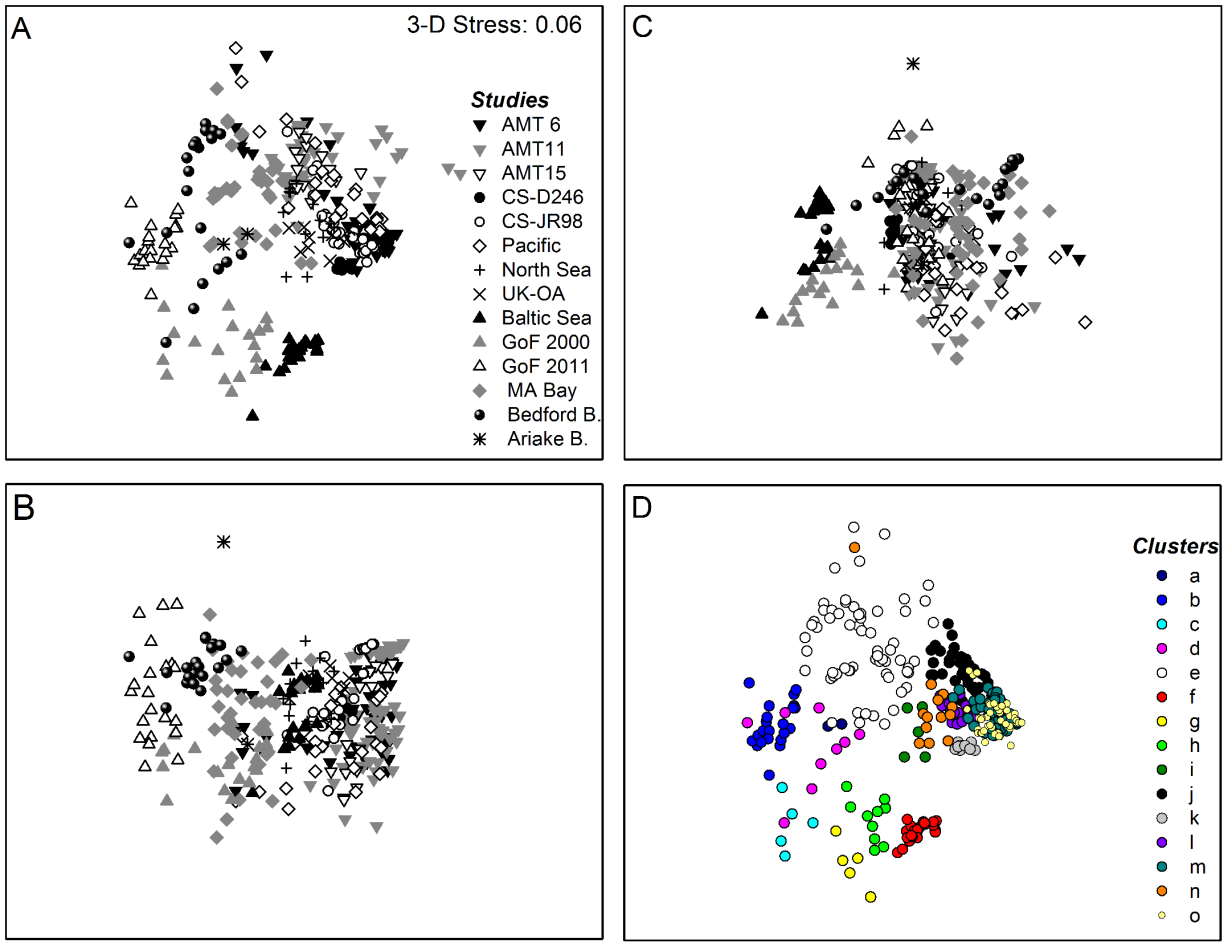
Three-dimensional non-metric Multidimensional Scaling (nMDS) ordination of the environmental conditions of all field campaigns. Panels (A), (B) and (C) are the x-y, x-z and y-z plane of the nMDS 3D ordination, respectively. (D) is the x-y plane where the symbols denote the various clusters as determined by cluster analysis in combination with similarity profile (SIMPER) tests. Clusters were significantly different from another (SIMPROF, *p*<0.005). For an overview of sample groupings according to these clusters see details in text and Table 4.

Cluster analysis in combination with a SIMPROF test generated 15 significantly different clusters (*p*<0.005) labelled alphabetically from a-o (*p*<0.005) across a range of Euclidean distances (representing dissimilarities between samples) from 0–4.4; these clusters often overlapped in the nMDS ordination of the Euclidean distance matrix derived from the environmental variables, indicating that there existed similarities in some of the environmental conditions between samples from different studies (Table 4, Fig. 4).

**Table 4:** Samples grouped according to similarities in environmental conditions as determined by cluster analysis and SIMPROF.

Cluster	Description	n
a	Ariake Bay + Massachusetts Bay	2
b	Gulf of Finland (SUPREMO11)	22
c	Gulf of Finland 2000 (mid-Apr to mid-May)	5
d	Bedford Basin (mid-Mar to end of Apr)	9
e	Bedford Basin (Feb to mid-Mar), MA Bay, North Sea CEND0811, AMT6 UPW and Pacific Ocean UPW	67
f	Baltic proper (SYNTAX2010)	22
g	Gulf of Finland 2000 mid May, mid and late Jul, early Aug	5
h	Gulf of Finland late May to early Jul, Aug to Oct	10
i	North Sea CEND0811 (Pen Field, South Pen Field, D366 (Falmouth, Southern North Sea, Helgoland)	5
j	Offshore DCM of AMT6, 11, 15 & Pacific Ocean, Celtic Sea JR98 DCM	49
k	Celtic Sea D246, AMT 6 European shelf edge surface	9
l	AMT6, 11 European shelf edge intermediate depths, Celtic Sea JR98 DCM, CEFAS0811 (North Dogger), D366 (Central North Sea)	17
m	AMT6 open ocean, surface & intermediate depths, AMT 11 open ocean intermediate depths, Pacific Ocean SPG surface, HNLC surface	54
n	Celtic Sea JR98 (Irish Sea), North Sea (D366 Falmouth, St George Straight, Mingulay, Bay of Biscay	12
o	AMT6, 11. 15 open ocean surface, D366 Central North Sea, Celtic Sea (JR98) surface	45

SPG South Pacific Gyre, HNLC High Nutrient Low Chlorophyll region, DCM Deep Chlorophyll Maximum; n is the number of samples per cluster.

**Figure 5:**
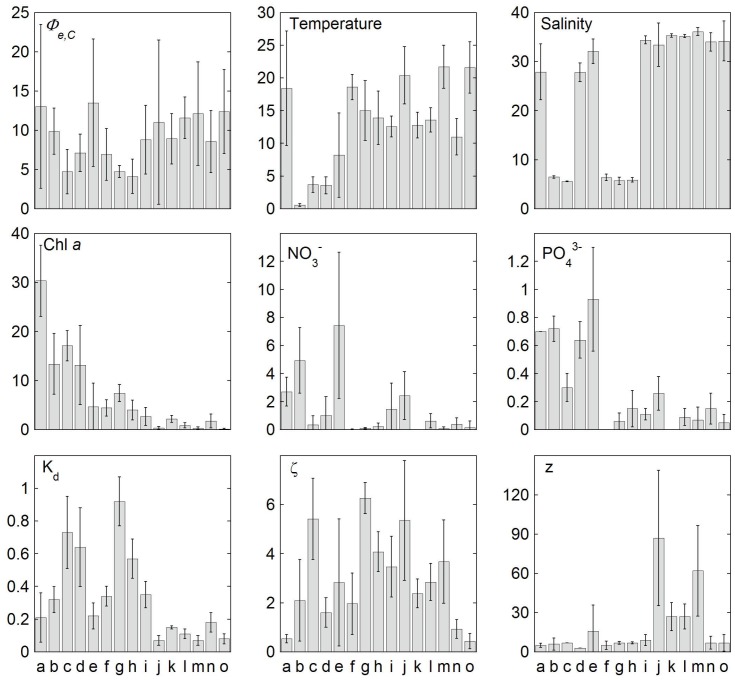
Variability in the electron requirement for carbon fixation (*Φ*_e,C_) and environmental conditions within different clusters of samples. Clusters were generated by cluster analysis combined with a SIMPROF test. For samples contained in each cluster see also Table 4. Values are means and error bars are standard deviations (with n of 2–67, see Table 4) of *Φ*_e,C_ (mol e^−^ mol C^−1^), temperature (°C), salinity, chlorophyll *a* (Chl *a*, mg m^−3^), nitrate and phosphate (µmol L^−1^), the vertical attenuation coefficient of photosynthetically available radiation (K_d_, m^−1^), optical depth ξ (dimensionless) and sampling depth (z, in meters).

Some of the resulting clusters corresponded to obvious biogeographic regions and/or seasonal (i.e. temperature dependent) groupings, while others represent more or less distinct water masses (Table 4). All the samples collected in the Baltic Sea and Gulf of Finland fell into biogeographically distinct clusters (b, c, f, g and h) characterised by low salinities (Fig. 5). The four Gulf of Finland clusters (b, c, g, h) clearly represented temporal changes in environmental conditions with samples in cluster b being characterised by extremely low temperatures during late winter (SUPREMO11 cruise) coupled with high nutrient availability. Low temperature and high nutrients set this cluster apart from spring cluster c with still relatively low temperatures(<5 °C) and clusters g and h, which contain all the summer and autumn samples characterized by warm temperatures (∼15 °C), low NO_3_^−^ availability and high *K_d_* and *ζ* values. Samples from the Baltic Sea proper (cluster f) were characterised by almost undetectable nutrient concentrations. Other clusters with samples representative of distinct regions include the North Sea cluster i and cluster k containing Celtic Sea samples and samples from the European shelf edge collected during AMT6. Like in the Baltic Sea, deplete nutrient concentrations in cluster k sets this cluster apart from cluster i.

Samples from Bedford Basin fell into two clusters: cluster d containing samples collected from mid-March to mid-May when chlorophyll concentrations increased and nutrient concentrations and water clarity dropped from previous levels that characterized samples from cluster e, which were collected in late winter (February to mid-March). Interestingly, cluster e also contained almost all of the Massachusetts Bay samples, some North Sea samples from frontal regions as well as AMT 6 and Pacific Ocean samples from the Humboldt and Benguela upwelling areas, respectively. This represents similarities in physico-chemical properties of these different water masses rather than biogeographical regions. During late winter, salinity in the ice covered Bedford Basin was similar to that in off shore waters, while chlorophyll and nutrient concentrations as well as optical properties matched those of upwelling/frontal regions; hence, their grouping together.

The remaining clusters (j, l, m, n, o) also contained samples from a variety of different cruises/regions. Samples collected from the deep chlorophyll maximum (DCM) in off shore regions during the AMT cruises, the Pacific Ocean cruise and the Celtic Sea (JR98) fell into one cluster (j), which was characterized by high temperatures as well as higher nutrient availability and ζ relative to the other offshore samples. Cluster l contained samples from intermediate depths in temperate waters on the European shelf and the shelf edge, which had relatively low temperatures. What set cluster l apart from cluster n, which also contained samples from the European shelf, was its overall greater optical depth. Open ocean samples with high water clarity and temperatures, as well as extremely low chlorophyll and nutrient concentrations fell into clusters m and o. While cluster m contained samples from intermediate depths collected during AMT6 and AMT11 and from the surface of the South Pacific Gyre (SPG) and HNLC region in the Pacific, cluster o was comprised of surface samples from the open ocean (all AMT cruises), central North Sea and Celtic Sea (JR98).

**Figure 6:**
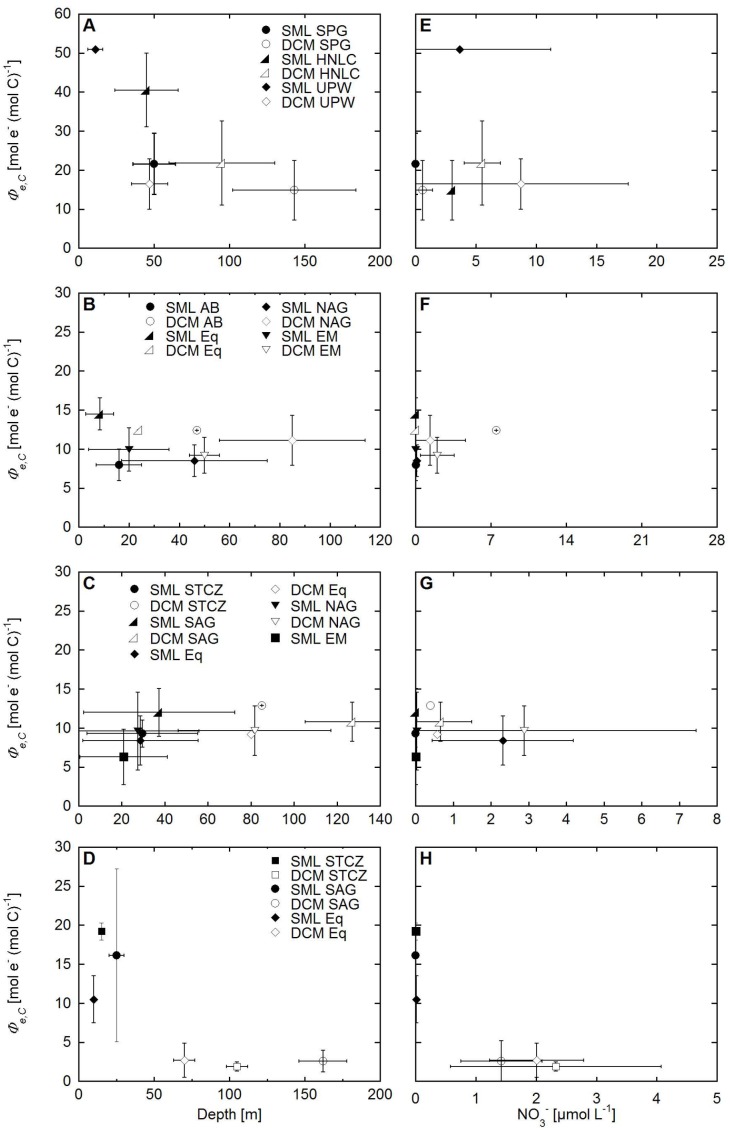
Mean electron requirement for carbon fixation (*Φ_e,C_*) across different biogeographical provinces for four cruises. *Φ_e,C_* versus depth (left panels) and NO_3_^−^ (right panels) are shown for the Pacific Ocean (A and E), AMT 6 (B and F), AMT 11 (C and G) and AMT 15 (D and H). Error bars are standard deviations with n = 3 to 14. SML denotes surface mixed layer, DCM the deep chlorophyll maximum, SPG is the South Pacific Gyre, HNLC High Nutrient Low Chlorophyll areas, UPW upwelling regions, SA South Africa, AB Angola Basin, Eq equator, NAG North Atlantic Gyre, EM Eastern Margin in the North East Atlantic, and STCS the Subtropical Convergence Zone. Note change in x- and y-axis scale.

Clearly, temperature, salinity, chlorophyll, nutrient concentrations and light availability were the main environmental factors responsible for the distinct grouping of samples into clusters. Given that cluster analysis in strongly stratified, off-shore waters resulted in distinct groupings corresponding to DCM, intermediate depth and surface samples and due to the pronounced effects of water column stratification on light and nutrient availability, we also plotted the mean *Φ_e,C_*, grouped according to samples from the surface mixed layer (SML) and DCM against depth and NO_3_^−^ for each biogeographic province sampled during the Pacific and AMT cruises (Fig. 6). As such, distinct differences in *Φ_e,C_* existed between the SML and DCM samples from the Pacific and AMT 15 cruise, where *Φ_e,C_* was much higher in the SML than at the DCM and the increase in *Φ_e,C_* coincided with a drop in nutrient availability or even depletion of nutrients in surface waters. During the AMT6 and AMT11 cruises, on the other hand, no such pronounced differences in *Φ_e,C_* and nutrient concentrations between SML and DCM was observed for the sampled locations in most of the biogeographic provinces.

In summary, there existed distinct groupings for many of the cruises and regions based on differences in environmental gradients within and between regions and water masses. This also confirms that user specific differences in methodology did not appear to have a systematic influence.

### Relationships between environmental conditions and *Φ_e,C_*

Clustering all available data based on the inherent environmental characteristics demonstrated that the dependence of *Φ_e,C_* on environmental gradients cannot be resolved at the level of the individual studies. Cluster analysis therefore enabled us to objectively identify how to pool data across the multidimensional environmental variable matrix (Table 4) to further examine variability of *Φ_e,C_*. Permutation tests conducted on the individual clusters (i.e. clusters a–q, Table 4) indeed showed that often multiple variables combined were significantly correlated with *Φ_e,C_* (BEST, *p*<0.05 or *p*<0.01), and that different variable combinations were driving *Φ_e,C_* in different clusters and regions (Table 5). These ‘best’ variable combinations were entered into multiple linear regression to derive algorithms for the prediction of *Φ_e,C_*, which, in many cases, resulted in significant relationships with *Φ_e,C_* (MLR, *p*<0.05) (Table 5). Significant relationships (*R^2^* <0.05) between *Φ_e,C_* and environmental variables existed in the Gulf of Finland (cluster b), Bedford Basin (cluster d), European shelf waters (cluster n) and offshore samples from intermediate depths (cluster m), surface waters (cluster n) and the deep chlorophyll maximum (DCM) (cluster j). Surprisingly, PO_4_^3−^, rather than NO_3_^−^ availability, was often part of the variable combinations showing the strongest association with *Φ_e,C_*, usually in addition to temperature and/or salinity. The strong relationships between *Φ_e,C_* and PO_4_^3−^ and lack thereof with NO_3_^−^ may be due to NO_3_^−^ concentrations being close to or below the detection limit in open ocean waters and, during the summer months, often also in shelf waters, which compromises the detection of relationships with *Φ_e,C_*. Thus, NO_3_^−^ availability may still be an important determinant of *Φ_e,C_* in these waters, but with PO_4_^3−^ being the only macronutrient left in our analysis, only the latter was pulled out. Despite the distinct clustering of samples from the frontal and upwelling regions, the Baltic Sea and European shelf edge, no significant MLR existed between *Φ_e,C_* and environmental variables in any of these regions (clusters e–i, k, l).

**Table 5:** Multivariate correlations and regressions between environmental variables and *Φ_e,C_* for different clusters and regions.

	BEST			MLR		
Cluster	ρ	p	Variables	Model	R^2^	p
a	-	-	-	-	-	-
b GoF	0.238	>0.05	T, S, K_d_, ζ	−3.82T+2.80S+3.01K_d_–1.12ζ	0.735	<0.01
c GoF	0.538	>0.05	S	n.s.	0.701	>0.05
d Bedford Basin	0.303	>0.05	T	−1.31T+11.78	0.710	<0.05
e Fronts and UPW	0.234	<0.01	S	n.s.	0.085	>0.05
f Baltic proper	0.135	>0.05	T	n.s.	0.103	>0.05
g GoF	0.430	>0.05	N, P, K_d_	n.s.	0.676	>0.05
h GoF	0.195	>0.05	S, PO_4_^3−^	n.s.	0.369	>0.05
i North Sea	0.539	>0.05	S, PO_4_^3−^, ζ	n.s.	0.965	>0.05
j DCM offshore	0.402	<0.01	T, S	−8.05T+8.89S−3.23	0.251	<0.01
k Celtic Sea, EUR shelf edge	−0.05	>0.05	NO_3_^−^, Chla	n.s.	0.150	>0.05
l EUR shelf edge	0.254	>0.05	ξ	n.s.	0.012	>0.05
m Interm. depths offshore	0.201	<0.05	S, N, P, Chla	−50.85S+4.25NO_3_^−^+0.675PO_4_^3−^ −11.81Chla+322.8	0.315	<0.01
n EUR shelf, North Sea	0.468	>0.05	T	6.40T −13.46	0.434	<0.05
o Surface offshore	0.190	>0.05	S, No_3_^−^, Chla	−4.09S −5.83NO_3_^−^+12.09Chla+32.59	0.269	<0.01
Biogeographic regions						
GoF (b c g h)	0.447	<0.01	T, S, NO_3_^−^, K_d_	−0.741T+14.8S+0.206NO_3_^−^ −2.69K_d_ −26.14	0.561	<0.01
Baltic + GoF (b c g h e)	0.236	<0.01	S, NO_3_^−^, Chla, K_d_, ζ	1.30S −5.21NO_3_^−^ −0.61Chla −2.53K_d_+0.77ζ+5.75	0.266	<0.01
Shelf (i k l n)	0.245	<0.01	T, NO_3_^−^, PO_4_^3−^, Chla, ζ	3.39T+1.44NO_3_^−^ −2.20PO_4_^3−^ −1.42Chla −0.57ζ–0.25	0.273	<0.05
Offshore SML (m o)	0.173	<0.05	NO_3_−, PO_4_^3−^, Chla	−5.97NO_3_^−^+9.62PO_4_^3−^+0.33Chla +11.35	0.083	<0.01
Offshore SML+DCM (j m o)	0.296	<0.01	T	−5.45T+37.51	0.090	<0.01
EUR Shelf/NE Atlantic	0.142	<0.05	T, PO_4_^3−^, Chla	0.04T −11.53PO_4_^3−^ −1.09Chla+10.99	0.207	<0.01
NW Atlantic Shelf	0.329	<0.01	T, S, NO_3_^−^, PO_4_^3−^	−0.57T+0.25S+1.36NO_3_^−^ −5.66 PO_4_^3−^+22.58	0.322	<0.01
Equatorial Atlantic	0.205	<0.05	S	0.47S −0.723	0.090	<0.05
South Atlantic	0.395	<0.01	NO_3_^−^, PO_4_^3−^, Chla	0.32NO_3_^−^ −13.07PO_4_^3−^+0.78Chla+6.20	0.213	<0.05
Pacific SPG +HNLC	0.233	>0.05	S, PO_4_^3−^, Chla	−25.27S −94.29PO_4_^3−^+29.29Chla+920	0.589	<0.01
Pacific SPG + HNLC + UPW	0.183	>0.05	S, PO_4_^3−^, Chla	17.53S+33.11PO_4_^3−^+14.6 Chla+1397	0.554	<0.01
Global	0.114	<0.01	T, NO_3_^−^, PO_4_^3−^	0.31T −0.49NO_3_^−^+6.34PO_4_^3−^+4.05	0.038	<0.01

Results of the BEST test are the variable combinations resulting in the highest correlation coefficient (ρ) between the resemblance matrices of the environmental data and *Φ_e,C_*. Abbreviations: GoF Gulf of Finland, UPW Upwelling region, SML surface mixed layer, DCM deep chlorophyll maximum, EUR European SPG South Pacific Gyre, HNLC High nutrient low chlorophyll area,. Cluster a was excluded from the BEST test and MLR due to its small sample size (n = 2).

To develop region-specific algorithms, we also grouped the clusters according to meaningful water masses and biogeographic regions resulting in 11 significant algorithms covering the Gulf of Finland, the Baltic Sea as a whole (i.e. including the Gulf of Finland), European shelf waters (including the Northeast Atlantic), Northwest Atlantic shelf waters, the equatorial Atlantic and the South Atlantic Ocean. Although some of these region-specific relationships exhibited a low *R^2^*, they were highly significant. The relationships between *Φ_e,C_* and environmental variables were strongest in the Gulf of Finland and the Pacific Ocean (*R^2^*<0.05), followed by the Northwest Atlantic shelf samples (i.e. Massachusetts Bay + Bedford Basin). Relationships for the Atlantic were much less pronounced (*R^2^* <0.22), albeit highly significant (*p*<0.01). Once again, PO_4_^3−^ rather than NO_3_^−^ availability appeared to play a greater role in these MLRs. On a global scale, MLR yielded an *R^2^* of 0.038, but with both NO_3_^−^ and PO_4_^3−^ contributing to the variability in *Φ_e,C_*.

## Discussion

### Reconciling electron transfer and carbon fixation

While empirical evidence demonstrates that fluorescence-based measures of the PSII photochemical efficiency, and hence ETRs, are linearly related to net or gross carbon fixation rates under many conditions [16], [47], [54], there are still considerable uncertainties about what drives *Φ_e,C_* as well as differences between NPP and GPP. Growth rate dependent differential allocation of fixed carbon and varying lifetimes of intermediate products may cause large discrepancies between NPP and GPP, which short term ^14^CO_2_ uptake measurements may not capture [19], [20]. While it is generally accepted that short-term ^14^C incubation techniques approximate GPP, Halsey et al. [19], [20] demonstrated that this is only the case in fast growing, nutrient-replete phytoplankton. In nutrient limited cells, on the other hand, short-term ^14^C incubations equate NPP or primary productivity rates somewhere between NPP and GPP. Failure to accurately quantify GPP will inevitably affect *Φ_e,C_*. Most data published in the past and also those used in our meta-analysis compared ETR to relatively short-term ^14^CO_2_ uptake rates, so that most *Φ_e,C_* values available to this day would provide a conversion from ETR to GPP or to something between NPP and GPP. Under most conditions, NPP differs from GPP by a factor of 2 to 2.5 [55], [19], [20], for which the current *Φ_e,C_* values cannot account. Conversion of ETR to NPP and assessments of the potential error in *Φ_e,C_* and subsequently NPP due to employing short term ^14^C incubations will require further investigation and is the focus of ongoing work.

Under ‘optimal’ growth (where NPP ≈ GPP), and accounting for electron sinks associated with nutrient reduction, the slope of the linear relationship between ETR and NPP should yield values for *Φ_e,C_* of 4–6 mol e^−^ (mol C)^−1^ [16]–[18]. To date, most FRRf-based studies use an electron requirement for carbon fixation of 5 mol e^−^ (mol C)^−1^ to convert ETRs to carbon fixation rates, assuming (i) that at least 4 e^−^ transported through PSII are required per O_2_ molecule produced and (ii) that 1–1.5 mol of O_2_ is produced for each mol CO_2_ fixed, i.e. the photosynthetic quotient takes values of 1–1.5 mol O_2_ (mol CO_2_)^−1^ [56]. Indeed, some studies here confirmed *Φ_e,C_* equal (or close to) 4–6 mol e^−^ (mol C)^−1^). However, the vast majority of estimates for *Φ_e,C_* are considerably higher, sometimes reaching extremes of >50 mol e^−^ (mol C)^−1^ (e.g. BIOSOPE Pacific Ocean Cruise) or alternatively *Φ_e,C_* of <5 mol e^−^ (mol C)^−1^ Gulf of Finland 2000 and AMT 15. Thus, for many cases, application of an assumed value of 4–6 mol e^−^ (mol C)^−1^ to FRR data would yield erroneous estimates of C-uptake (within the limitations of the ^14^C-uptake itself for quantifying C-uptake).

Values of *Φ_e,C_* >5 mol e^−^ (mol C)^−1^ have been observed previously and potentially reflect processes that act to decouple ETRs from C-fixation, such as photorespiration [32], chlororespiration via a plastid terminal oxidase (PTOX) [33], [34] and Mehler reaction [32]. We return to this issue in the following sections. In contrast, values of *Φ_e,C_* <5 mol e^−^ (mol C)^−1^ are more difficult to reconcile with biophysical and physiological processes, potentially indicating the magnitude of remaining methodological discrepancies in deriving *Φ_e,C_*, such as incorrect assumptions concerning the value or variability of *n_PSII_* (see below), inadequate spectral correction or remaining non-systematic errors in both carbon fixation and fluorescence estimates. There were two studies with a high proportion of *Φ_e,C_* values <5 mol e^−^ (mol C)^−1^: the AMT15 cruise (Hickman et al. unpublished) and the Gulf of Finland 2000 study. Unlike during the AMT15 cruise where <5 mol e^−^ (mol C)^−1^ occurred only in samples from the DCM, we could not identify any consistency in the occurrence of such low values that could be related to sample handling and processing in the Gulf of Finland 2000 data. Thus, the high proportion of low *Φ_e,C_* values in the Gulf of Finland might point to systematic errors in the ETR calculations, i.e. in the component values for *σ_PSII_'*, *n_PSI_*_I_ and/or E, values that are often assumed (or not well measured). Whilst values of *σ_PSII_'* measured by FRRf have been shown to match independent bio-optical measurements well [46], we have to assume that the bio-optical instrumentation used to quantify both E and *σ_PSII_'* have been appropriately calibrated. In the Gulf of Finland [29], values of *σ_PSII_'* typically range from ca. 150–300 A^2^ quantum^−1^, which is on the lower end of the range of values expected for assemblages dominated by diatoms, dinoflagellates, cryptophytes and cyanobacteria (data not shown), but still within the range commonly observed for such species [38]. Absolute underestimations of *σ_PSII_'* as a result of erroneous instrument calibrations are therefore unlikely a significantly contributing factor. However, in cases when cyanobacteria dominate, ETRs may be underestimated due to the saturating light blue LED pulse being inefficient at driving reaction centre closure, resulting in low *Φ_e,C_* values. Furthermore, assumption of a constant *n_PSII_* will introduce errors due to taxonomic variability in physiological traits [46]. Moreover, phytoplankton cells tend to change the size of their photosynthetic units in response to light and nutrient availability [57]–[59] and vertical mixing [60]. Hence *n_PSII_* can vary among species by more than a factor of four, from 0.0010 to 0.0042 mol RC (mol Chl*a*)^−1^ [46]. Raateoja et al. [29] assumed a constant *n_PSII_* of 0.002 mol RC (mol Chl*a*)^−1^, which is representative of the eukaryote phytoplankton community observed in their study (mostly diatoms, dinoflagellates and cryptophytes; but not typically when cyanobacteria dominate. Consequently, increasing assumed values of *n_PSII_* in the study of Rateeoja et al. [29] to account for the presence of cyanobacteria [8], [46] would increase ETRs and hence potentially *Φ_e,C_* to values >5 mol e^−^ (mol C)^−1^.

We assessed the potential error in *Φ_e,C_* due to the use of a constant *n_PSII_* in some of the data sets presented in this study by comparing *Φ_e,C_* based on a constant *n_PSI_*_I_ with *Φ_e,C_* values calculated from ETR where *n_PSII_* was either measured via oxygen flash yields (Bedford Basin data) or calculated using a fluorescence based algorithm [7] (CEND0811, UK-OA D366). These comparisons showed that a constant *n_PSII_* may lead to an underestimate of *Φ_e,C_* by 28–47% (Fig. S2, Table S1 in Appendix S1), which could thus, at least in part, explain the low *Φ_e,C_* in the Gulf of Finland data. The *n_PSII_* values estimated for the North Sea spanned a range of 0.0018 to 0.0056 mol RC (mol chl*a*)^−1^, which is comparable with the range observed for eukaryotic and prokaryotic phytoplankton [46], [47], [58], but higher, by ca. a factor of 2, than *n_PSII_* directly measured by Moore et al. [11] in European shelf waters at a similar time of year. Clearly further direct measurements of *n_PSII_* and validation of algorithms proposed for estimating the value of this variable [7] are required before a more robust assessment of the error in calculated *Φ_e,C_* which is associated with *n_PSII_* can be achieved. Such measurements are the focus of ongoing work.

Other sources of discrepancies between electron transfer and carbon fixation may stem from differences among protocols for ETR-light response curves (rapid light curves vs. steady state light curves) [61], deviations in the timescales of *in situ* ETR and laboratory ^14^C-photoynthesis measurements [43], [61], discrepancies in the E_K_ values derived from ETR and CO_2_ uptake [52]–[54], or the effects of water column structure and subsequent changes in light availability on the ‘shape’ of ETR light response curves [8]. For the data included in the present study and the associated differences in methodology with regard to ETR calculations (*σ_PSII_'*, *n_PSI_*_I_) and ^14^C incubation techniques, we estimated that, in worst case scenarios, *Φ_e,C_* may be over- or underestimated by as much as 53% (data not shown). A detailed assessment of these methodological differences between techniques and studies is beyond the scope of this study, and we refer the reader to a previous comprehensive reviews of these issues [8], [38]. However, we note that variability in estimated values of *Φ_e,C_* was lower than that observed across all studies (Fig. 2) for the two new studies included here, which were performed under the most controlled conditions (SYNTAX2010 and North Sea CEND0811), i.e. were ETR and CO_2_ fixation were simultaneously measured on the same sample. Thus, the extent to which the extreme values and/or variability in *Φ_e,C_*, which is observed in some other studies, represents remaining errors in either carbon fixation or ETR estimates remains unclear.

### Environmental regulation of *Φ_e,C_*

Data presented in this study focused almost exclusively on the apparent effect of readily measurable environmental variables on *Φ_e,C_*. Information on phytoplankton community composition could not be routinely included and we assumed that any change in taxonomy was inherently accounted for in the environmental descriptors. Indeed, environment often leads to distinct structural differences of the photosynthetic apparatus and acclimation responses amongst phytoplankton from different biogeographic regions [34], [62]–[67] and, towards unique, often consistent fluorescence "signatures" of PSII [10], [11], [38], [68]–[70]. Future assessments of the inter- and intraspecific variability in *Φ_e,C_* will, of course, be necessary to fully resolve the mechanisms responsible for changes in *Φ_e,C_* and the derived algorithms (Table 5), which are purely based on statistical models and should therefore not be interpreted from a mechanistic perspective. From the relatively few available studies on phytoplankton cultures grown under various conditions to date, *Φ_e,C_* usually does not exceed values of ∼25 mol e^−^ (mol C)^−1^ as opposed to estimates of up to 54 mol e^−^ mol C^−1^ from natural communities (reviewed in Suggett et al. [8]). Lower maximal values for phytoplankton cultures could indicate a less extreme range of growth environments tested in the laboratory as compared to natural conditions, such as, the high-light, low-nutrient conditions of the surface open ocean that are difficult to reproduce in the laboratory. Furthermore, laboratory studies often examine cells that are in ‘steady state’ growth whereas natural communities may persist under (rapidly) changing environmental conditions. In the latter case, non-steady state conditions could lead to a strong and transient uncoupling of electron transfer and carbon fixation [10], [71], [72], which would suggest that the primary source of variation in *Φ_e,C_* is the environment rather than taxonomic variability. The effect of growth rate on our ability to measure GPP by short-term ^14^C incubations under natural conditions may further contribute to the wider range of *Φ_e,C_* values from field observations relative to culture-based *Φ_e,C_* [19], [20].

Considerable variability of *Φ_e,C_* existed not only spatially but also temporally. Given the large range of *Φ_e,C_* for the entire data set, it is notable that mean *Φ_e,C_* in all but one of the time series studies (Massachusetts Bay) never exceeded 10 mol e^−^ (mol C)^−1^. The greater variance in the Massachusetts Bay time series study could, on the one hand, have been related to its longer duration (9 month) relative to the other time series (1.5–6 month) and, on the other hand, to its topography; while the Gulf of Finland, Bedford Basin and Ariake Bay, are all surrounded by land forming at least partially land-enclosed basins, Massachusetts Bay is relatively exposed and more strongly influenced by exchange of water with the Atlantic Ocean. Nevertheless, seasonal changes appear to influence the variability in *Φ_e,C_* although many areas are still under-sampled.

In the Gulf of Finland, for example, vertical mixing and the depth of the surface mixed layer change across different seasons [73]–[75] and with it the optical and physicochemical properties of the water column, that is, light and nutrient availability of the extant phytoplankton community [29]. The clustering of the Gulf of Finland samples - into a late winter (b), spring (c), summer (g) and summer/early autumn (h) mirrors these physicochemical changes. However, the highly dynamic nature of this system makes the development of predictive algorithms for the entire Baltic Sea (including the Gulf of Finland) challenging. The best predictors of *Φ_e,C_* for the whole region were temperature, salinity and PO_4_^3−^, resulting in a relatively weak (*R* ^2^ = 0.266) albeit highly significant relationship. For the Gulf of Finland on its own, however, the relationships were much stronger (*R^2^* = 0.561, *p*<0.01), indicating that the Gulf of Finland should perhaps be treated as its own region.

Distinct temporal changes in environmental gradients also existed in Bedford Basin, where initial correlations (Table 2) showed significant relationships of *Φ_e,C_* with salinity, which may have both indirect (via alterations of density and water column stratification) and direct (osmotic) effects on *Φ_e,C_*. The combination of low nutrients coupled with sudden high light availability due to elevated freshwater discharge after snow melt and ice breakup could cause an up-regulation of electron, ATP and/or reductant-consuming pathways (e.g. photorespiration and Mehler reaction). Conceivably, similar changes in salinity could also directly affect *Φ_e,C_* by causing osmotic stress or an increase in respiration of the extant phytoplankton community [76]. Bedford Basin phytoplankton community composition often shifts from diatoms and dinoflagellates to chlorophytes after the snow melt (Suggett & Forget unpublished) [77], matching the increase of *Φ_e,C_*. Thus, in Bedford Basin multiple environmental factors (salinity, temperature, stratification) were likely at play, potentially exerting light and osmotic stress on the extant, but changing, phytoplankton community [77]–[80]. Resolving the underlying mechanisms responsible for seasonal changes in *Φ_e,C_* in remains a challenge and will require teasing apart daylight hour effects from temperature effects and other physicochemical variables.

Many samples from Massachusetts Bay and other open ocean areas fell into one cluster with the Bedford Basin data (Table 4, Fig. 5). The Bedford Basin spring cluster (d) was characterized by high chlorophyll concentrations, whereas samples in the late winter cluster (e) had low chlorophyll concentrations and temperatures more similar to those in frontal and upwelling regions of the North Sea, and the Atlantic and Pacific Oceans. The wide range of environmental gradients observed in these clusters may be the reason why MLR did not return any significant relationships between *Φ_e,C_* and environmental variables. Furthermore, such relationships may not necessarily be linear [81] and other models will have to be tested in the future.

Most of the other studies, also extended across multiple biogeographic provinces (sensu Longhurst [82]) or distinct environments water masses (SML/DCM), and although there seemed to be consistent overlaps between nutrient deplete regions and areas of high *Φ_e,C_* (Fig. 3), the absolute nutrient concentration was often not correlated with *Φ_e,C_* (Table 2). Important examples are the open ocean regions, highlighting how environmental forcing can confound establishment of strong relationships between *Φ_e,C_* and measurable environmental variables at the basin scale: *Φ_e,C_* often declined with increasing depth and towards the nutricline; even so, *Φ_e,C_* was often not significantly correlated with measured nutrient concentration most likely reflecting differences in nutrient availability between the gyres, HNLC areas and coastal upwelling regions. Additionally, nutrient stocks are not necessarily a good index of the level of nutrient stress within a population, which will depend rather on the overall (re-)supply rates of the nutrient relative to the demands of the extant community. In the iron and nitrogen deplete SPG [12] and the low-iron HNLC region of the Pacific Ocean [83], *Φ_e,C_* declined when NO_3_^−^ concentrations were above the detection limit (usually at the deep chlorophyll maximum). Apart from the extreme outlier of *Φ_e,C_*>50 mol e^−^ (mol C)^−1^, highest *Φ_e,C_* values were observed in the upwelling region and the gyre corresponding to samples from a low-NO_3_^−^ surface water lens with high irradiances. Beneath this surface water lens, NO_3_^−^ increased while light levels dropped and *Φ_e,C_* declined.

Iron and nitrogen limitation may be expected to influence the photosynthetic apparatus in different ways, with iron deficiency causing a preferential decline in iron rich-cellular components (e.g. PS I, PSII and cytochrome b_6_f) [56], [84]–[86]. Thus, different forms of nutrient limitation can induce differences in the fluorescence signatures of the resident phytoplankton community [67], [72] and subsequently in *Φ_e,C_*. Iron limited phytoplankton in HNLC regions, for instance, my express the chlorophyll-binding protein IsiA, which reduces the apparent PSII photosynthetic efficiency and primary productivity rates normalized to chlorophyll [87], [88]. The latter would cause *Φ_e,C_* to increase. Nutrient limitation in combination with high-light stress in surface waters may hence have been responsible for the highest *Φ_e,C_* obtained in these regions, suggesting a high degree of uncoupling between electron transfer and carbon fixation. Similarly, variations in nitrogen assimilation and nitrogen fixation require large amounts of ATP also leading to an uncoupling of electron transport from carbon fixation [67], [89], [90].

### Conclusions

Conversions of FRRf-based ETR estimates to carbon-specific rates of NPP depend on our ability to accurately predict *Φ_e,C_* for given environmental condition. This present study shows, for the first time, the extent of variability of *Φ_e,C_*, albeit within the past methodological constraints of accurate quantification of both ETRs and NPP. Most data on *Φ_e,C_* available to this day are based on short-term ^14^C incubations which, depending on algal growth rates, may capture GPP rather than NPP. Further studies are needed to fully resolve the discrepancies between *Φ_e,C_* derived from GPP or NPP in both cultures and natural communities. Nevertheless, our work shows that some of the variability in *Φ_e,C_* can be linked to environmental variables that are routinely measured. Given the observed variability, it is highly unlikely that a global approach or algorithm can be produced which captures a high proportion of the variance in *Φ_e,C_*. However, with the present study we provide firm evidence that such algorithms can in fact be generated for biogeographic regions and distinct water masses, bringing us closer than ever to predicting carbon uptake from ETRs. Independent validation of these algorithms is still required, and, we may expect they will be refined as more data accumulate. Importantly, our study provides methodology for future data collection and integration to improve *Φ_e,C_* algorithms. Clearly, developing standardized protocols would greatly facilitate inter-comparisons of studies and reduce some of the potential error due to methodological differences. Assessing the role of phytoplankton taxonomy, development of non-linear models and testing of the present algorithms on novel, independent data represent necessary future steps to improve the level of accuracy.

One of the major remaining challenges in utilising these algorithms is defining the (biogeographic) regions, while keeping in mind that their boundaries might actually shift in space and time. Hence caution is still required in widespread application of the current algorithms. Even so, the appearance of clear patterns and proof of predictive power of environmental variables in the present article provides strong support and much improved confidence for successful conversion of ETRs to carbon fixation rates at a much greater spatial and temporal resolution than current ^14^C fixation approaches.

## Supporting Information

Figure S1 **Principal component analysis including the environmental data, location data and methodological information (A) and with the methodological differences excluded from the analysis (B).**
(TIF)

Figure S2 **Comparison of** ***Φ_e,C_*** **(in mol e^−^ (mol C)^−1^) calculated with an** ***n_PSII_*** **= 0.0020 mol RC (mol chl** ***a*** **)^−1^ and where** ***n_PSII_*** **was measured with oxygen flash yields (Bedford Basin) or by FRR fluorometry according to Oxborough et al.** [**11**] **(UK-OA D366 and North Sea CEND0811).** Bold line represents the regression equation for all three studies combined: *Φ_e,C_* constant *n_PSII_* = 0.617× (*Φ_e,C_* measured *n_PSII_*)+2.765 (*R^2^* = 0.807, n = 110, p<0.05). Regression coefficients are shown in Table S1 in Appendix S1.
(TIF)

Appendix S1 **Results of PCA including methodological and location variables and assessment of the effect of variable nPSII on Phie,C.**
(DOCX)

## References

[1] Lindeman RL (1942) The trophic dynamic aspect of ecology. Ecology 23: 399–418.

[2] Field CB, Behrenfeld MJ, Randerson JT, Falkowski PG (1998) Primary production of the biosphere: Integrating terrestrial and oceanic components. Science 281: 237–240. 9657713 10.1126/science.281.5374.237

[3] Carr M-E, Friedrichs AM, Schmeltz M, Aita MN, Antoine D, et al (2006) A comparison of global estimates of marine primary production from ocean color. Deep Sea Res II 533: 741770 doi:10.1016/j.dsr2.2006.01.028

[4] Longhurst A, Sathyendranath S, Platt T, Caverhill C (1995) An estimate of global primary production in the ocean from satellite radiometer data. J Plankton Res 17: 1245–1271.

[5] Behrenfeld MJ, Boss E, Siegel DA, Shea DM (2005) Carbon-based ocean productivity and phytoplankton physiology from space. Global Biogeochem Cycles 19: GB1006 doi:10.1029/2004GB002299

[6] Schreiber U, Neubauer C, Schuwa U (1993) PAM fluorometer based on medium-frequency pulsed Xe-flash measuring light: A highly sensitive new tool in basic and applied photosynthesis research. Photosynth Res 36: 65–72. 24318799 10.1007/BF00018076

[7] Oxborough K, Moore MC, Suggett D, Lawson T, Chan HG, et al (2012) Direct estimation of functional PSII reaction centre concentration and PSII electron flux on a volume basis: a new approach to the analysis of Fast Repetition Rate fluorometry (FRRf) data. Limnol Oceanogr Meth 10: 142–154.

[8] Suggett DJ, Moore M, Geider RJ (2011) Estimating aquatic productivity from active fluorescence measurements. In: Suggett DJ, Prášil O, Borowitzka MA, editors. Chlorophyll *a* fluorescence in aquatic sciences: methods and applications. Springer Dordrecht. pp. 103–127.

[9] Falkowski PG, Kolber ZS (1995) Variations in Chlorophyll Fluorescence Yields in Phytoplankton in the World Oceans. Austr J Plant Physiol 22: 341–355.

[10] Moore MC, Suggett DJ, Holligan PM, Sharples J, Abrahams ER (2003) Physical controls on phytoplankton physiology at a shelf front: a fast repetition-rate fluorometer based field study. Mar Ecol Prog Ser 259: 29–45.

[11] Moore MC, Suggett DJ, Hickman AE, Kim Y-N, Tweddle JF, et al (2006) Phytoplankton photoacclimation and photoadaptation in response to environmental gradients in a shelf sea. Limnol. Oceanogr. 5: 936–949.

[12] Behrenfeld MJ, Kolber ZS (1999) Widespread iron limitation of phytoplankton in the South Pacific Ocean. Science 283: 840–843. 9933166 10.1126/science.283.5403.840

[13] MacIntyre HL, Cullen JJ (2005) Using cultures to investigate the physiological ecology of microalgae. In: Andersen RA, editor. Algal culturing techniques. Elsevier Academic Press, New York. pp. 287–236.

[14] Venrick EL, Beers, JR, Heinbokel JF (1977) Possible consequences of containing microplankton for physiological rate measurements. J Experimental Mar Biol Ecol 26: 55–76.

[15] Fogg GE, Calvario-Martinez O (1989) Effects of bottle size in determinations of primary productivity by phytoplankton. Hydrobiologia 173: 89–94.

[16] Genty B, Briantais J-M, Baker NR (1989) The relationship between the quantum yield of photosynthetic electron transport and quenching of chlorophyll fluorescence. Biochim Biophys Acta 990: 87–92.

[17] Edwards GE, Baker NR (1993) Can CO_2_ assimilation in maize leaves be predicted accurately from chlorophyll fluorescence analysis? Photosynth Res 37: 89–102. 24317706 10.1007/BF02187468

[18] Suggett DJ, MacIntyre HL, Kana TM, Geider RJ (2009) Comparing electron transport with gas exchange: parameterising exchange rates between alternative photosynthetic currencies for eukaryotic phytoplankton. Aquatic Microbial Ecol 56: 147–162.

[19] Halsey KH, Milligan AJ, Behrenfeld MJ (2010) Physiological optimization underlies growth rate-dependent chlorophyll-specific gross and net primary production. Photosynth Res 103: 125–137. 20066494 10.1007/s11120-009-9526-z

[20] Halsey KH, Milligan AJ, Behrenfeld MJ (2011) Linking time-dependent carbon-fixation efficiencies in Dunaliella tertiolecta (Chlorophyceae) to underlying metabolic pathways. J Phycol 47: 66–76. 27021711 10.1111/j.1529-8817.2010.00945.x

[21] Boyd PW, Aiken J, Kolber ZS (1997) Comparisons of radiocarbon and fluorescence based (pump and probe) measurements of phytoplankton photosynthetic characteristics in the Northeast Atlantic Ocean. Mar Ecol Prog Ser 149: 215–226.

[22] Corno G, Letelier RM, Abbott MR, Karl DA (2006) Assessing primary production variability in the north Pacific subtropical gyre: a comparison of fast repetition rate fluorometry and ^14^C measurements. J Phycol 42: 51–60.

[23] Kromkamp JC, Dijkman NA, Peene J, Simis SGH, Gons HJ (2008) Estimating phytoplankton primary production in Lake Ijsselmeer (The Netherlands) using variable fluorescence (PAM-FRRF) and ^14^C-uptake techniques. Eur J Phycol 43: 327–344.

[24] Suggett DJ (2000) Variability of phytoplankton productivity rates in the Atlantic Oceans observed using the Fast Repetition Rate fluorometer. University of Southampton, United Kingdom, Doctoral Thesis, 184 pp.

[25] Suggett DJ, Moore MC, Marañón E, Omachi C, Varela RA, et al (2006) Photosynthetic electron turnover in the tropical and subtropical Atlantic Ocean. Deep Sea Res II 53: 1573–1592.

[26] Hickman (2007) The photophysiology and primary productivity of phytoplankton within the deep chlorophyll maximum. University of Southampton, School of Ocean and Earth Sciences, Doctoral Thesis, 237pp.

[27] Pemberton KL, Clarke R, Joint I (2006) Quantifying uncertainties associated with the measurement of primary production. Mar Ecol Prog Ser 322: 51–59.

[28] Smyth TJ, Pemberton KL, Aiken J, Geider RJ (2004) A methodology to determine primary production and phytoplankton photosynthetic parameters from fast repetition rate fluorometry. Journal of Plankton Research 26: 1337–1350.

[29] Raateoja N, Seppälä J, Kuosa H (2004) Bio-optical modelling of primary production in the SW Finnish coastal zone, Baltic Sea: fast repetition rate fluorometry in Case 2 waters. Mar Ecol Prog Ser 267: 9–26.

[30] Melrose DC, Oviatt CA, O'Reilly JE, Berman MS (2006) Comparisons of fast repetition rate fluorescence estimated primary production and ^14^C uptake by phytoplankton. Mar Ecol Prog Ser 311: 37–46.

[31] Tripathy SC, Ishizaka J, Fujiki T, Shibata T, Okamura K, et al (2010) Assessment of carbon- and fluorescence-based primary productivity in Ariake Bay, southwestern Japan. Estuarine Coastal Shelf Sc 87: 163–173.

[32] Badger MR, von Caemmerer S, Ruuska S, Nakano H (2000) Electron flow to oxygen in higher plants and algae: rates and control of direct photoreduction (Mehler reaction) and rubisco oxygenase. Phil Trans R Soc Lond D 355: 1433–1446. 10.1098/rstb.2000.0704 PMC1692866 11127997

[33] Bailey S, Mehlis A, Mackey KRM, Cardol P, Finazi G, et al (2008) Alternative photosynthetic electron flow to oxygen in marine *Synechococcus* . Biochim Biophys Acta 1777: 269–276. 18241667 10.1016/j.bbabio.2008.01.002

[34] Mackey KRM, Paytan A, Grossman AR, Bailey S (2008) A photosynthetic strategy for coping in a high-light, low-nutrient environment. Limnol Oceanogr 53: 900–913.

[35] Holmes JJ, Weger HG, Turpin DH (1989) Chlorophyll *a* fluorescence predicts total phytosynthetic electron flow to CO_2_ or NO_3_ ^−^/NO_2_ ^−^ under transient conditions. Plant Physiol 91: 331–337. 16667020 10.1104/pp.91.1.331 PMC1061995

[36] Estévez-Blanco P, Cermeño P, Espiñeira M, Fernández E (2006) Phytoplankton photosynthetic efficiency and primary production rates estimated from fast repetition rate fluorometry at coastal embayments affected by upwelling (Rías Baixas, NW of Spain). J Plankton Res 28: 1153–1165.

[37] Behrenfeld MJ, Prášil O, Babin M, Bruyant F (2004) In search of a physiological basis for covariations in light-limited and light-saturated photosynthesis. J Phycol 40: 4–25.

[38] Suggett DJ, Moore MC, Hickman AE, Geider RJ (2009) Interpretation of fast repetition rate (FRR) fluorescence: signatures of phytoplankton community structure versus physiological state. Mar Ecol Prog Ser 376: 1–19.

[39] Kirk JTO (2010) Light and photosynthesis in aquatic ecosystems, 3^rd^ edition. Cambridge: Cambridge University Press. 662 p.

[40] Suggett DJ, Kraay,G, Holligan P, Davey M, Aiken J, et al (2001) Assessment of photosynthesis in a spring cyanobacterial bloom by use of a fast repetition rate fluorometer. Limnol Oceanogr 46: 802–810.

[41] Kolber ZS, Prášil O, Falkowski PG (1998) Measurements of variable chlorophyll fluorescence using fast repetition rate techniques: defining methodology and experimental protocols. Biochim Biophys Acta 1367: 88–106. 9784616 10.1016/s0005-2728(98)00135-2

[42] Suggett DJ, Oxborough K, Baker NR, MacIntyre HL, Kana TM, et al (2003) Fast repetition rate and pulse amplitude modulation chlorophyll a fluorescence measurements for assessment of photosynthetic electron transport in marine phytoplankton. Eur J Phycol 38: 371–384.

[43] Kromkamp JC, Forster RM (2003) The use of variable fluorescence measurements in aquatic ecosystems: differences between multiple and single turnover protocols and suggested terminology. Eur J Phycol 38: 103–112.

[44] Suggett DJ, Maberly SC, Geider RJ (2006) Gross photosynthesis and lake community metabolism during the spring phytoplankton bloom. Limnol Oceanogr 51: 2064–2076.

[45] Cullen JJ, Davis RF (2003) The blank can make a big difference in oceanographic measurements. Limnol Oceangr Bull 12: 29–35.

[46] Suggett DJ, MacIntyre HL, Geider RJ (2004) Evaluation of biophysical and optical determinations of light absorption by photosystem II in phytoplankton. Limnol Oceanogr Meth 2: 316–332.

[47] Kolber ZS, Falkowski PG (1993) Use of active fluorescence to estimate phytoplankton photosynthesis *in situ* . Limnol Oceangr 38: 1646–1665.

[48] Ting CS, Owens G (1994) The effects of excess irradiance on photosynthesis in the marine diatom *Phaeodactylum tricornutum* . Plant Physiol 106: 763–770. 12232368 10.1104/pp.106.2.763 PMC159585

[49] Laney SR, Letelier RM, Desiderio RA, Abbott MR, Kiefer DA, et al (2001) Measuring the natural fluorescence of phytoplankton cultures. J Atmos Ocean Technol 18: 1924–34.

[50] Milligan AJ, Aparicio UA, Behrenfeld MJ (2011) Fluorescence and nonphotochemical quenching responses to simulate vertical mixing in the marine diatom *Thalassiosira weissflogii* . Mar Ecol Prog Ser 448: 67–78.

[51] Lewis MR, Smith JC (1983) A Small Volume, Short-Incubation-Time Method for Measurement of Photosynthesis As A Function of Incident Irradiance. Mar Ecol Prog Ser 13: 99–102.

[52] MacIntrye HL, Kana TM, Anning T, Geider RJ (2002) Photoacclimation of photosynthesis irradiance response curves and photosynthetic pigments in microalgae and cyanobacteria. J Phycol 38: 17–38.

[53] Clarke KR, Somerfield PJ, Gorley RN (2008) Testing of null hypotheses in exploratory community analyses: similarity profiles and biota-environment linkage. J Exp Mar Biol Ecol 366: 56–69.

[54] Baker NR, Oxborough K (2004) Chlorophyll fluorescence as a probe of photosynthetic productivity. In: Papageorgiou GC, Govindjee, editors. Chlorophyll fluorescence: a signature of photosynthesis. Springer, Dordrecht. pp. 65–85.

[55] Williams PJ le B, Robinson C, Søndergaard M, Jespersen A-M, Bentley TL, et al (1996) Algal 14C and total carbon metabolism. 2. Experimental observations with the diatom *Skeletonema costatum* . J Plankton Res 18: 1961–1974.

[56] Laws EA (1991) Photosynthetic quotients, new production and net community production in the open ocean. Deep Sea Res 30: 143–167.

[57] Falkowski PG, Owens TG (1980) Light-shade adaption - two strategies in marine phytoplankton. Plant 66: 592–595. 10.1104/pp.66.4.592 PMC440685 16661484

[58] Dubinsky Z, Falkowski PG, Wyman K (1986) Light harvesting and utilization by phytoplankton. Plant Cell Physiol 27: 1335–1349.

[59] Berges JA, Charlebois DO, Mauzerall DC, Falkowski PG (1996) Differential effects of nitrogen limitation on photosynthetic efficiency of photosystem I and II in microalge. Plant Physiol 110: 689–696. 12226211 10.1104/pp.110.2.689 PMC157765

[60] Kromkamp J, Limbeek M (1993) Effect of short-term variation in irradiance on light-harvesting and photosynthesis of the marine diatom *Skeletonema costatum*: a laboratory study simulating vertical mixing. J General Microbiol 139: 2277–2284.

[61] Serôdio J, Vieira S, Cruz S, Coelho H (2006) Microphytobenthos vertical migratory photoresponse as characterized by light response curces. Photosynth Res 90: 29–43. 17111236 10.1007/s11120-006-9105-5

[62] Sunda WG, Huntsman SA (1995) Photosynthetic architecture differs in oceanic and coastal phytoplankton. Mar Chem 50: 189–206.

[63] Scanlan DJ (2003) Physiological diversity and niche adaptation in marine *Synechococcus* . Adv Microb Physiol 47: 1–64. 14560662 10.1016/s0065-2911(03)47001-x

[64] Strzepek RF, Harrison PJ (2004) Photosynthetic architecture differs in coastal and oceanic diatoms. Nature 431: 689–692. 15470428 10.1038/nature02954

[65] Cardol P, Bailleul B, Rappaport F, Derelle E, Beal D, et al (2008) An original adaptation of photosynthesis in the marine green alga *Ostreococcus* . Proc Natl Acad Sci USA 105: 7881–7886. 18511560 10.1073/pnas.0802762105 PMC2409423

[66] Bibby TS, Zhang Y, Chen M (2009) Biogeography of photosynthetic light-harvesting genes in marine phytoplankton. PloS ONE. 4: e4601. 10.1371/journal.pone.0004601 PMC2644788 19240807

[67] Grossman AR, Mackey KRM, Bailey S (2010) A perspective on photosynthesis in the oligotrophic oeans: hypotheses concerning alternate routes of electron flow. J Phycol 46: 629–634.

[68] Behrenfeld MJ, Worthington K, Sherrell RM, Chavez FP, Strutton P, et al (2006) Controls on tropical Pacific Ocean productivity revealed through nutrient stress diagnostics. Nature 442: 1025–1028. 16943835 10.1038/nature05083

[69] Behrenfeld MJ, Halsey KH, Milligan AJ (2008) Evolved physiological responses of phytoplankton to their integrated growth environment. Phil Trans R Soc Lond B 363: 2687–2703. 18487129 10.1098/rstb.2008.0019 PMC2606763

[70] Liu S-W, B-S Qiu (2012) Different responses of photosynthesis and flow cytometric signals to iron limitation and nitrogen source in coastal and oceanic *Synechococcus* strains (Cyanophyceae). Mar Biol 159: 519–532.

[71] Masojídek J, Grobbelaar JU, Pechar L, Kobližek M (2001) Photosystem II electron transport and oxygen production in natural waterblooms of freshwater cyanobacteria during a diel cycle. J Plankton Res 23: 57–66.

[72] Wagner H, Jakob T, Wilhelm C (2006) Balancing the energy flow from captured light to biomass under fluctuating light conditions. New Phytol 169: 95–108. 16390422 10.1111/j.1469-8137.2005.01550.x

[73] Tamminen T, Andersen T (2007) Seasonal phytoplankton nutrient limitation patterns as revealed by bioassays over Baltic Sea gradients of salinity and eutrophication. Mar Ecol Prog Ser 340: 121–138.

[74] Lessin G, Lips I, Raudsepp U (2007) Modelling nitrogen and phosphorus limitation on phytoplankton growth in Narva Bay, south-eastern Gulf of Finland. Oceanologia 49: 259–276.

[75] van Beusekom JJE, Mengedoht D, Augusting CB, Schilling M, Boersma M (2009) Phytoplankton, protozooplankton and nutrient dynamics in the Bornholm Basin (Baltic Sea) in 2002–2003 during the German GLOBEC Project. Int J Earth Sci 98: 251–260.

[76] Flameling IA, Kromkamp J (1984) Responses of respiration and photosynthesis of S*cenedesmus protuberans* Fritsch to gradual and steep salinity increases. J Plankton Res 16: 1781–1791.

[77] Li WKW, Harrison WG, Head EJH (2006) Coherent assembly of phytoplankton communities in diverse temperate ocean ecosystems. Proc Royal Soc B Biol Sci 273(1596): 1953–1960. 10.1098/rspb.2006.3529 PMC1634774 16822757

[78] Wu Y, Peterson IK, Tang CCL, Platt T, Sathyendranath S, et al (2007) The impact of sea ice on the initiation of the spring bloom on the Newfoundland and Labrador Shelves. J Plankton Res 29: 509–514.

[79] Wu Y, Platt T, Tang CCL, Sathyendranath S (2008) Regional differences in the timing of the spring bloom in the Labrador Sea. Mar Ecol Prog Ser 355: 9–20.

[80] Zhai L, Platt T, Tang C, Sathyendranath S, Walls RH (2011) Phytoplankton phenology on the Scotian Shelf. ICES J Mar Sci 68: 781–791.

[81] Napoléon C, Claquin P (2012) Multi-parametric relationships between PAM measurements and carbon incorporation, an *in situ* approach. PLoS ONE 7: e40284. 22911698 10.1371/journal.pone.0040284 PMC3401225

[82] Longhurst AR (1998) Ecological geography of the sea. Academic Press, 398 p.

[83] Bonnet S, Guieu C, Bruyant F, Prasil O, Van Wambeke F, et al (2008) Nutrient limitation of primary productivity in the Southeast Pacific (BIOSOPE cruise). Biogeosciences 5: 215–225.

[84] Greene RM, Geider RJ, Falkowski PG (1991) Effect of iron limitation on photosynthesis in a marine diatom. Limnol Oceanogr 36: 1772–1782.

[85] Greene RM, Geider RJ, Kolber Z, Falkowski PG (1992) Iron-induced changes in light harvesting and photochemical energy conversion processes in eukaryotic marine algae. Plant Physiol 100: 565–575. 16653030 10.1104/pp.100.2.565 PMC1075596

[86] Geider RJ, LaRoche J, Greene RM, Olaizola M (1993) Response of the photosynthetic apparatus of *Phaeodactylum tricornutum* (Bacillariophyceae) to nitrate, phosphate, or iron starvation. J Phycol 29: 755–766.

[87] Schrader PS, Milligan AJ, Behrenfeld MJ (2011) Surplus photosynthetic antennae complexes underlie diagnostics of iron limitation in a cyanobacterium. PLoS ONE 6: e18753. 21533084 10.1371/journal.pone.0018753 PMC3080375

[88] Ryan-Keogh TJ, Macey AI, Cackshutt AM, Moore MC, Bibby TS (2012) The cyanobacterial chlorophyll-binding-protein IsiA Acts to increase the in vivo effective absorption cross section of PSI under iron limitation. J Phycol 48: 145–154. 27009659 10.1111/j.1529-8817.2011.01092.x

[89] Levitan O, Rosenberg G, Setlik I, Setlokova E, Grigel J, et al (2007) Elevated CO_2_ enhances nitrogen fixation and growth in the marine cyanobacterium *Trichodesmium* . Global Change Biol 13: 531–538.

[90] Levitan O, Kranz SA, Spungin D, Prášil O, Rost B, et al (2010) Combined effects of CO_2_ and light on the N2-fixing cyanobacterium Trichodesmium IMS101: A mechanistic view. Plant Physiol 154: 346–356. 20625002 10.1104/pp.110.159285 PMC2938161

