# Supplementary figures and images for: Predicting the Electron Requirement for Carbon Fixation in Seas and Oceans
Source: PLoS One. 2013 Mar 13;8(3):e58137. doi: 10.1371/journal.pone.0058137 (PMC3596381; doi:10.1371/journal.pone.0058137)

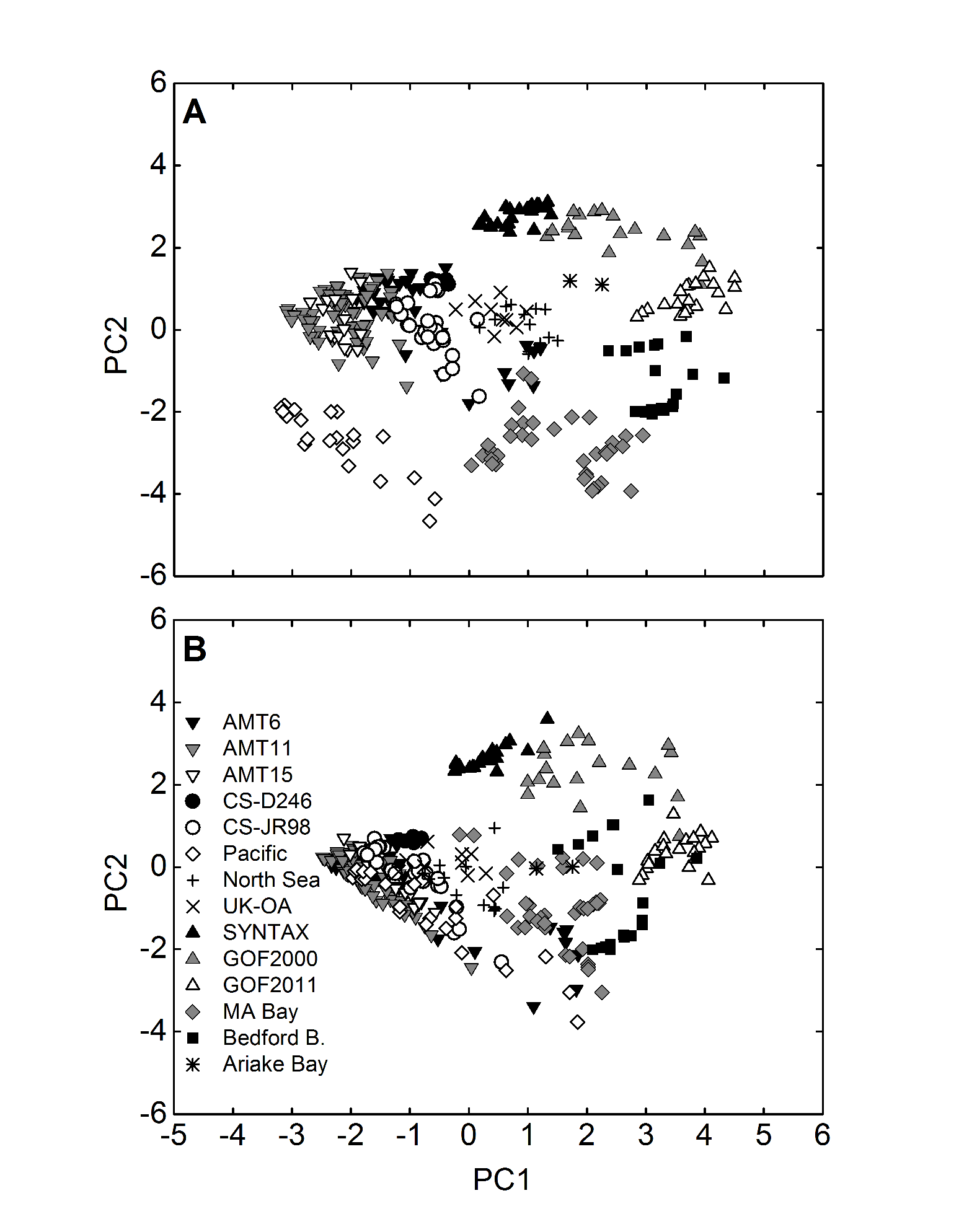

Supplement: Figure S1 — Principal component analysis including the environmental data, location data and methodological information (A) and with the methodological differences excluded from the analysis (B). (TIF) [file pone.0058137.s001.tif]

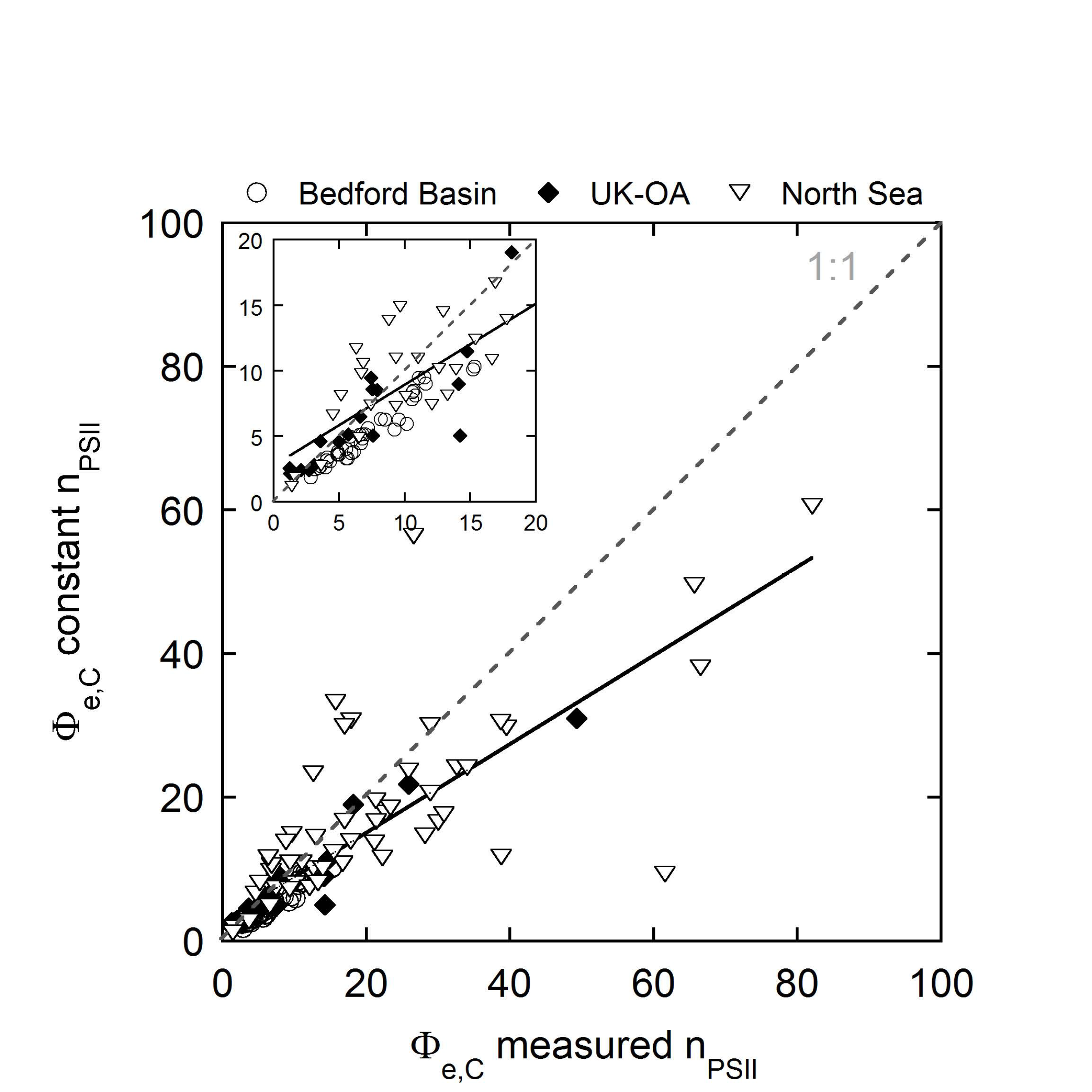

Supplement: Figure S2 — Comparison of Φe,C (in mol e− (mol C)−1) calculated with an nPSII = 0.0020 mol RC (mol chl a )−1 and where nPSII was measured with oxygen flash yields (Bedford Basin) or by FRR fluorometry according to Oxborough et al. [11] (UK-OA D366 and North Sea CEND0811). Bold line represents the regression equation for all three studies combined: Φe,C constant nPSII = 0.617× (Φe,C measured nPSII)+2.765 (R2 = 0.807, n = 110, p<0.05). Regression coefficients are shown in Table S1 in Appendix S1. (TIF) [file pone.0058137.s002.tif]
