# Supplementary material for: Predicting the Electron Requirement for Carbon Fixation in Seas and Oceans
Source: PLoS One. 2013 Mar 13;8(3):e58137. doi: 10.1371/journal.pone.0058137 (PMC3596381; doi:10.1371/journal.pone.0058137)
Supplement: Appendix S1 — Results of PCA including methodological and location variables and assessment of the effect of variable nPSII on Phie,C. (DOCX) [file pone.0058137.s003.docx]

**Appendix S1**

Effect of methodological and location information on the clustering of samples

There existed considerable differences in methodological approaches between the different studies included in the meta-analysis, particularly with regard to the assumptions made about the number of photosystem II (*n_PSII_*), spectral corrections applied to the absorption cross section of photosystem II (*σ_PSII_*) and the ratio of E:E_K_ (see main text for further explanations). Furthermore, inclusion of location (latitude, longitude and depth) and seasonal data (Julian day) may directly influence the outcome of the cluster analysis. To ensure that similarities in environmental conditions rather than methods and location/season drive the clustering of samples, some of the analyses were performed with and without methodological information.

Inclusion of the methodological information and location/seasonal data resulted in a more pronounced separation of samples along the principal components (PCs) while the overall patterns were similar (Fig. S1), indicating that environmental gradients are the main drivers of the clustering. Thus, differences in methods do not contribute much to the variability between samples, justifying the exclusion of these variables in the main analyses.

Effect of variable *n_PSII_* on *Φ_e,C_*

One common difference between the different methods for calculating electron transfer rates (ETRs) lay in the assumptions made about *n_PSII_* or the expression used to derive latter. Values of *n_PSII_* were either measured by oxygen flash yields, set constant (0.002-0.003 mol RCII (mol Chl)^-1^) or derived via algorithms published in Kolber and Falkowski [1] and Oxborough et al. [2]. Thus, the potential effect of differences in *n_PSII_* on *Φ_e,C_* caused by use of different approaches was assessed in three of the studies included in the meta-analysis. According to this comparison, a constant *n_PSII_* may lead to considerable underestimates in *Φ_e,C_* relative to *n_PSII_* values measured by oxygen flash yields or derived according to Oxborough et al. [2] (Table S1, Fig. S2).

REFERENCES

[1] Kolber ZS, Falkowski PG (1993) Use of active fluorescence to estimate phytoplankton photosynthesis *in situ*. Limnol Oceangr 38: 1646-1665.

[2] Oxborough K, Moore MC, Suggett D, Lawson T, Chan HG, Geider RJ (2012) Direct estimation of functional PSII reaction centre concentration and PSII electron flux on a volume basis: a new approach to the analysis of Fast Repetition Rate fluorometry (FRRf) data. Limnol Oceanogr Meth 10: 142-154.

**Supporting Information Table S1**. Slope, intercept and coefficient of determination (R^2^) for comparisons between *Φ_e,C_* (in mol e^-^ (mol C)^-1^) calculated with an ETR where *n_PSII_* = 0.0020 mol RC (mol chl*a*)^-1^ and where *n_PSII_* was measured with oxygen flash yields (Bedford Basin) or by FRR fluorometry according to Oxborough et al. [2] during the UK-OA D366 and North Sea CEND0811 cruises.

| Study | Slope | Intercept | R^2^ | n |
| --- | --- | --- | --- | --- |
| Bedford Basin | 0.720 | 0.067 | 0.936 | 40 |
| UK-OA D366 | 0.636 | 1.902 | 0.898 | 19 |
| North Sea CEND 0811 | 0.528 | 6.461 | 0.508 | 51 |
